# Concentration-dependent HAT/ET mechanism of the reaction of phenols with 2,2-diphenyl-1-picrylhydrazyl (dpph˙) in methanol†

**DOI:** 10.1039/d2ra01033j

**Published:** 2022-03-15

**Authors:** Paweł Przybylski, Adrian Konopko, Piotr Łętowski, Katarzyna Jodko-Piórecka, Grzegorz Litwinienko

**Affiliations:** University of Warsaw, Faculty of Chemistry Pasteura 1 02-093 Warsaw Poland litwin@chem.uw.edu.pl; Polish Academy of Science, Institute of Experimental Biology Pasteura 3 02-093 Warsaw Poland

## Abstract

The reaction of a 2,2-diphenyl-1-picrylhydrazyl radical (dpph˙) with phenols carried out in alcohols is a frequently used assay for estimation of the antiradical activity of phenolic compounds. The rates of reactions of dpph˙ with five phenols (ArOH: unsubstituted phenol, 4-hydroxyacetophenone, two calix[4]resorcinarenes and baicalein) measured in methanol indicate the different kinetics of the process for very diluted phenols compared to their non-diluted solutions. This effect was explained as dependent on the ratio [ArO^−^]/[ArOH] and for diluted ArOH corresponds to an increased contribution of much faster electron transfer (ET, ArO^−^/dpph˙) over the Hydrogen Atom Transfer (HAT, ArOH/dpph˙). Simplified analysis of the reaction kinetics resulted in estimation of *k*^ET^/*k*^HAT^ ratios for each studied ArOH, and in calculation of the rate constants *k*^ET^. Described results are cautionary examples of how the concentration of a phenol might change the reaction mechanism and the overall kinetics of the observed process.

## Abstract 2

Concentration dependent contribution of hydrogen atom transfer and electron transfer to the overall kinetics of reaction of phenols with a 2,2-diphenyl-1-picrylhydrazyl radical in methanol.

## Graphical abstract

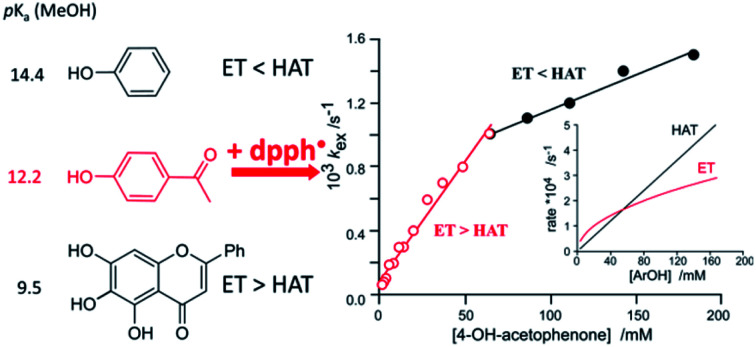

## Introduction

The stable 2,2-diphenyl-1-picrylhydrazyl radical, abbreviated as DPPH or dpph˙, is broadly employed for quick assessment of the radical scavenging abilities of natural and synthetic compounds. The methods are based on the monitoring of dpph˙ decoloration:

1 ArOH + dpph˙ → ArO˙ + dpph-H; overall *k*^S^

**Chart 1:**
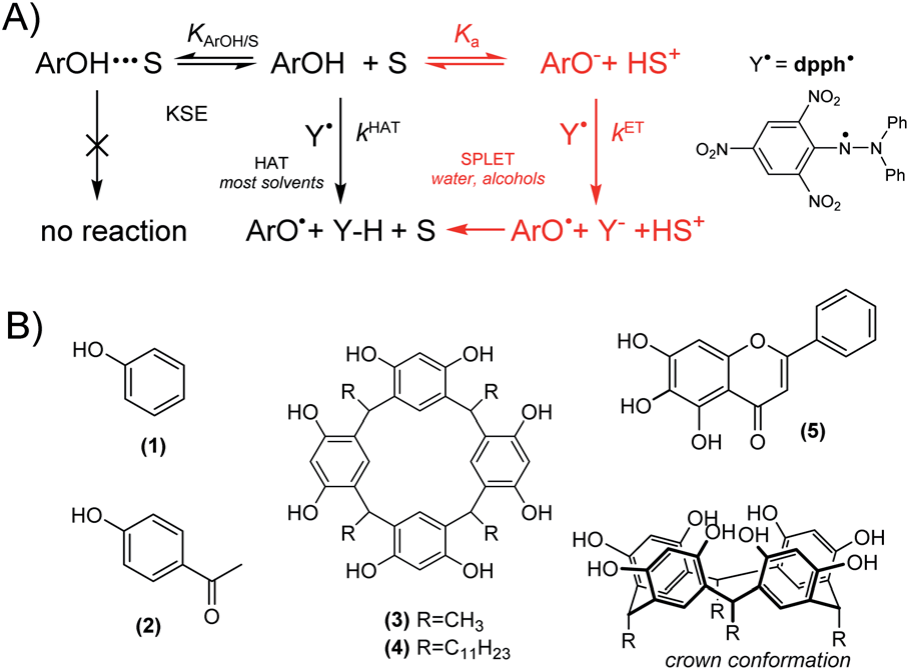
(A) General scheme of reaction of phenol (ArOH) with dpph˙ including kinetic solvent effect (KSE, left), HAT (central) or SPLET mechanisms (right, shown in red). (B) Structures of phenols 1–5, with crown conformation of 3 and 4.

In hydrocarbons and other non-polar solvents, reaction 1 proceeds as Hydrogen Atom Transfer (HAT). Since dpph˙ radicals are about 3 orders of magnitude less reactive than peroxyl radicals,^1^ reaction 1 can be easily employed for studies on reactivity of phenols and the kinetic solvent effect (KSE)^1,2^ (see Chart 1A), and even for estimation of O–H bond strengths. However, in ionization supporting solvents (water and alcohols) the HAT mechanism is “contaminated” by electron transfer (ET) from the ionized fraction of ArOH. This two-step mechanism is described as Sequential Proton-Loss Electron Transfer (SPLET),^3,4^ with *k*^ET^ >> *k*^HAT^, see Chart 1A, and even traces of ArO^−^ causing an enormous increase of overall rate of reaction 1.^2^ Mixed mechanism in alcohols excludes dpph˙ as mimetic to peroxyl radicals but, regardless of this controversy,^5^ reaction 1 is one of the most frequently used colorimetric assays employed for quick assessment of the antiradical abilities of natural and synthetic compounds, with more than 40 thousand papers published during the last decade (SCOPUS, keywords “dpph” and “antioxidant”).

In contrast to simple kinetic measurements, typical non-kinetic assays are based on a titration of dpph˙ solution with phenol (or mixture of phenol extracts) in order to determine IC_50_ parameter, *i.e.*, the concentration of phenol sufficient to scavenge 50% of the radicals present in the sample. However, reaction 1 can be (and usually is) reversible,^6^ because the bond dissociation enthalpy, BDE, in dpph-H (78.9 kcal mol^−1^)^6^ is lower than BDE_O–H_ for majority of phenols, and IC_50_ measurements can be misleading. Another problem was noticed by Foti *et al.* who reported that for some phenols reaction 1 exhibits non-integer order in [ArOH].^4–7^ Such confusing phenomena needs to be further explored, and we selected series of ArOH accommodating two opposing features: enhanced acidity and a measurable reactivity (within stopped-flow time scale) toward dpph˙. The proper selection of compounds was not trivial because the introduction of an electron-withdrawing group into ArOH enhances its acidity but also causes an increase in the strength of the O–H bond. Here we present the results obtained for: phenol (1), 4-hydroxyacetophenone (2), *C*-methylcalix[4]-resorcinarene (3), *C*-undecylcalix[4] resorcinarene (4), and baicalein (5, the only natural compound within this series, being also an interesting example of ArOH with a strongly acidic catechol moiety), see Chart 1B.

## Results and discussion

The reaction was monitored in neat methanol or, in order to suppress phenol ionization, in methanol acidified with acetic acid (AcOH). Experimental, pseudo-first-order rate constants, *k*_exp_, were determined for series of increasing [ArOH] being always in stoichiometric excess over dpph˙, and bimolecular rate constant (*k*^S^) was obtained from the straight-line equation:

2 *k*_exp_ = *k*^S^ [ArOH] + const

where the intercept (sometimes also denoted as *k*_0_)^1^ includes the self-decay of the radical which is not dependent on the ArOH concentration. In order to avoid the effect of reversibility of reaction 1 (*vide supra*), the very initial rates of reaction were measured (conversion of dpph˙ was less than 5–10%).

**Fig. 1:**
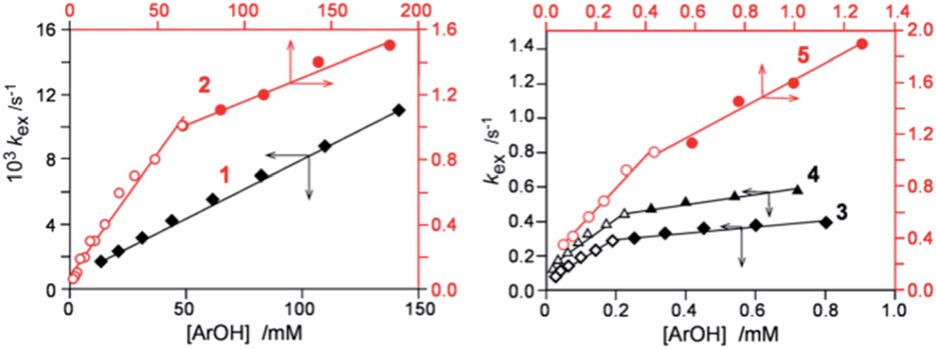
Plots of *k*_ex_*versus* [ArOH] for dpph˙ + phenols in neat methanol. Left panel: less reactive phenols 1 and 2. Right panel: much faster reacting phenols 3–5.

Fig. 1 indicates that 1 is the only phenol for which a straight-line dependence of *k*_exp_ on [ArOH] was obtained within the whole [ArOH] range. For 2–5, after the initial linear increase in *k*_exp_ against increasing [ArOH] there is a break in the trend, and the overall plot is not linear. This means that the rate law is more complex and the species other than ArOH are involved in the rate determining step. Foti *et al.*^4*b*^ studied the reaction of quercetin (QH_2_) with dpph˙ in methanol/water, and interpreted non-integer order (*k*_exp_ ∼ [QH_2_]^0.4^) as resulting from the reversible formation of π-stacked pre-reaction complex of quercetin anion with dpph˙, followed by fast ET:^4*b*^

3 QH^−^+ dpph˙ ⇆ [QH^−^/dpph˙] → QH˙ + dpph^−^

A similar mechanism was also described for curcumin/dpph˙ pair in ethanol^4*d*^ whereas for phenols with OH group internally H bonded to the N-base a formation of the contact ion pair (ET to dpph˙) was proposed:^4*c*^

4 ArOH + dpph˙ ⇆ [ArOH˙^+^/dpph^−^] → ArO˙ + dpph-H

**Fig. 2:**
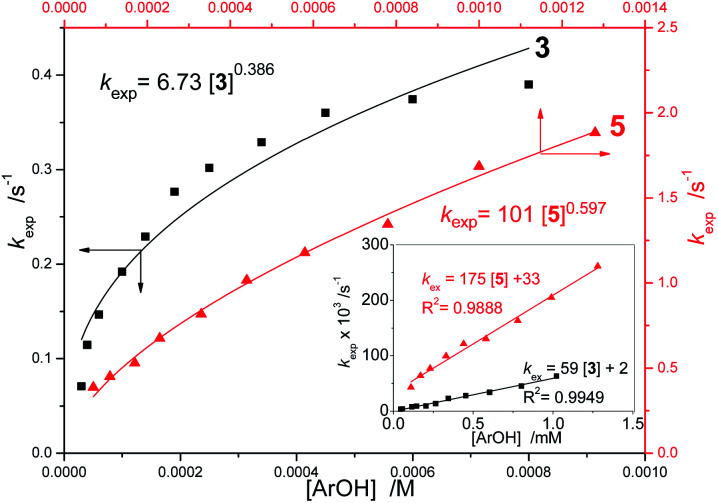
Plots of *k*_exp_ against [3] and [5] for reaction with dpph˙ in neat methanol with non-linear fit. Inset: plot of *k*_ex_*versus* [3] and [5] for reaction carried out in acidified methanol (10 mM AcOH) with linear fit.

Non-linear fitting of our data to a function *k*_exp_ = *a*[ArOH]^*b*^ gave reaction orders 0.38–0.60 with respect to concentration of 2–5, however, the goodness of fitting for 3 is rather moderate, with residuals as high as up to ±10%, see Fig. 2, S13, S25 and S30.† After addition of AcOH, the linear relationships (eqn (2)) were obtained‡‡Kinetic data from the inset can be also fitted for non-integer order model *k*_exp_ = ^*a*^[ArOH]^*b*^, giving artificial (in our opinion) *b* = 0.76 ± 0.10. For reaction of 5 with dpph˙ in methanol containing 100 and 1000 mM acetic acid the calculated *b* are scattered from 0.63 to 0.36 because even very small deviation of single experimental point produce substantial decrease of *b*, than can be either an error or an effect of mixed HAT/ET mechanism. Therefore, for acidified systems we did not push the reaction order into nonintegral orders in [ArOH]. Moreover, data for non-acidifiers 1 (straight line in Fig. 1A) can also be fitted to non linear function, giving reaction order 0.83. for the whole concentration range, see Fig. 2 and ESI,† proving the mixed HAT/SPLET mechanisms. Therefore, we limited our calculations of *k*^S^ for apparently straight line sectors of *k*_exp_*vs.* [ArOH] plots, below the inflection point, as presented in Fig. 1, and the results are listed in Table 1.

Bimolecular rate constants, *k*^S,^^a^ for reactions of dpph˙ with phenols 1–5 in neat and acidified^b^ methanol^c^

**Table:** 

phenol	1	2	3	4	5
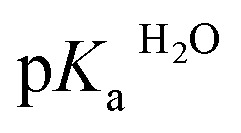 d	9.99	7.87	8.82/10.8/11.7	—	5.4/9.8/11.3
p*K*_a_^MeOH^^e^	14.4	12.2	13.2	—	9.5

a Max. errors ± 20%, see ESI for statistical parameters calculated for eqn (2) for each analyzed system.

b In mM.

c Parameters for methanol: HB accepting ability *β*^H^_2_ = 0.41,^8^ relative permittivity *ε*_r_ = 32.7, and autoprotolysis constant p*K*_SH_ = 17.2.^9^ For AcOH *β*^H^_2_ is assumed as 0.42 (the same value as for propionic acid).

d Values p*K*_a_ for 1, 2^10^ and 5^11^ in water, p*K*_a1–3_ for 3 in 1 : 1 water/methanol.^12^

e p*K*_a1_ in MeOH were calculated from the correlation: p*K*_a_ (MeOH) = 1.08 p*K*_a_ (H_2_O)+ 3.66 (*R* = 0.991).^13^ AcOH, with p*K*_a_ = 9.63 in methanol,^13^ is still stronger acid than phenols 1–4 but has almost the same acidity as 5. The pH window for water is 0–14, and for methanol 1.8–17.2.^9^

**Table:** 

[AcOH]^b^	*k* ^S^ a/(M^−1^ s^−1^)				
0	0.073	0.016	1100	1400	1900
10	0.011	0.011	56	77	210
100	0.010	0.012	53	66	37
1000	0.006	0.017	57	46	23

The value of *k*^S^ for reaction of 1 with dpph˙ is in a reasonable agreement with our previously published 0.04 M^−1^ s^−1^,^3*a*^ with some deviations that can be ascribed to a great sensitivity of the kinetics of the processes carried out in neat, non-buffered systems.§§To remove traces of phenols/stabilizers and other compounds that could contaminate the kinetics, we distilled methanol over a small amount of dpph˙ and a few beads of ion-exchange resin. BDE_O–H_ for 1 is 87.2 kcal mol^−1^ (ref. 6) or 88.2 kcal mol^−1^,^14^ and for 2 is 90.3 kcal mol^−1^,^14^ and ΔBDE = BDE_ArO–H_ − BDE_PhOH_ suggests that *k*^HAT^ for 2 should be 10–25 times smaller than for 1, as it can be predicted from eqn (5).¶¶This empirical equation is valid for non-hindered phenols reacting with dpph˙ radical in hydrocarbons (pure HAT mechanism, with no KSE). In other solvents some small differences can be observed due to different ability of ArOH and PhOH to form H bond with solvent, see discussion in footnote 34 in ref. 1. Predicted differences in *k*^S^ can be even bigger, because 2, with strong EW substituent, will be better HB donating agent than unsubstituted phenol 1. ^1^

5 log *k*_ArOH/dpph˙_ = −0.33 + 0.35(−ΔBDE)

However, only 5-fold (instead of 25-fold) difference in *k*^S^ indicates that *k*^HAT^ is partially compensated with a greater participation of the SPLET mechanism for 2 than for 1. Interestingly, addition of AcOH causes a decrease in *k*^S^ for 1, but not for 2 (even in methanol acidified with 1 M AcOH the parameter *k*^S^ was the same as in neat MeOH). This observation does not exclude SPLET because 2 is a relatively strong acid and its concentration is moderately high. Using *K*_a_ for 2 in methanol (footnote *e* in Table 1), we obtained [2^−^] = 0.56 × 10^−7^ M and 1.8 × 10^−7^ M for 5 mM and 50 mM solution of 2, respectively (this concentration range was used for calculation of *k*^S^). Furthermore, in this particular case acetic acid can slightly accelerate the completion of SPLET by fast protonation of dpph^−^ formed after ET from 2^−^ to dpph˙ (p*K*_a_ of dpph-H is 8.54 (ref. 15) or 8.59 (ref. 16) in methanol/water 1 : 1). Therefore, the equilibrium 2^−^ + dpph˙ ⇆ 2˙ + dpph^−^ will be shifted to the right. For reactive phenols this effect is kinetically not significant, but for a slowly and reversibly reacting electron deficient 2, the transfer of H^+^ from AcOH to dpph^−^ drives the reaction to the products (2˙/dpph-H). We also measured *k*^S^ for 1 and 2 reacting with dpph˙ in buffered methanol/water (1 : 1) at pH 5.4 and 7.4. Both compounds react faster than in neat methanol, confirming the role of deprotonation in the reaction mechanism. Surprisingly, at pH 7.4 both phenols, 1 and 2, react with almost the same rate, *k*^7.4^ is 0.74 ± 0.07 M^−1^ s^−1^ for 1 and 0.66 ± 0.12 M^−1^ s^−1^ for 2, whereas at pH 5.4 phenol 2 is a bit more reactive (*k*^5.4^ = 0.25 ± 0.01 M^−1^ s^−1^) than 1 (*k*^5.4^ = 0.19 ± 0.03 M^−1^ s^−1^). At pH 5.4 the plots of *k*_exp_*vs.* [2] are linear, but some deviations from linearity occur at pH 7.4 (see ESI†).

Phenols 3–5 are 10^5^ times more reactive than 1 and 2, therefore, much smaller concentrations (<1 mM) were used for measurements, and the presence of 10 mM AcOH (large excess) causes a 100-fold suppression of *k*^s^ for 5, and *ca.* 20-fold decrease for cyclic tetramers 3 and 4. A 10% better reactivity of 4 over 3 can be explained as an effect of a crown conformation of 4,^12^ with internal hydrogen bonds within the upper rim, see Chart 1B, facilitating the stabilization of a radical (although we cannot exclude other effects related to different conformations and causing small differences in acidity of both compounds).

Foti *et al.* used a model of π-stacking pre-reaction complex (eqn (3)) to explain a non-linear, concentration-dependent kinetic behavior of quercetin but in the same work a similar peculiarity was observed also for catechin, which does not form such complex.^4*b*^ Our results confirm that the mixed order of reaction is a more general phenomenon caused by HAT/ET competition and supported by ArOH/ArO^−^ ratio. The rate law is:

6 −d[dpph˙]/d*t* = *n* (*k*^HAT^[ArOH] + *k*^ET^[ArO^−^])[dpph˙]

For diluted solutions, the reaction order is very close to 1.0 (a straight line in Fig. 1). From the comparison of the reactions rates carried out for two different concentrations [ArOH]_1_ and [ArOH]_2,_ the proportion *k*_exp1_/*k*_exp2_ (pseudo-first order conditions) is obtained:7
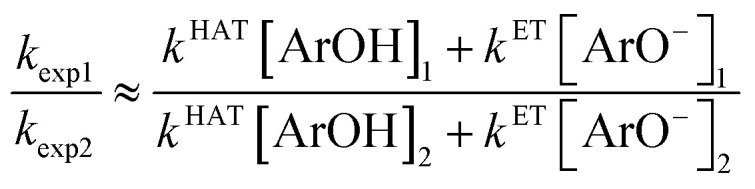


Eqn (7) can be solved after introducing 
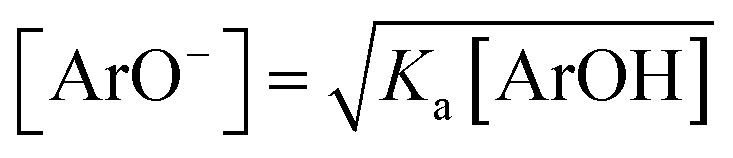
, with *K*_a_ values in methanol taken from Table 1, and separation of the variables, see ESI.† By introducing *k*_exp_ and [ArOH] pairs from straight-line sections of the plots of eqn (2), we obtained *k*^ET^/*k*^HAT^ ratio listed in Table 2. Such estimation gives the opportunity to compare the overall ET/HAT rates, without any additional knowledge about a formation of dpph˙/ArOH complexes.

**Table:** Reduction potentials in water at pH 7 (in mV, *vs.* NHE), *k*^ET^/*k*^HAT^ ratio, and *k*^ET^ (in M^−1^ s^−1^) for phenols 1, 2, 3, and 5

phenol	*k* ^ET^/*k*^HAT^	*k* ^ET^	*E* ^0^
1	1.5 × 10^6^	8.8 × 10^3^	970^a^
2	1.4 × 10^5^	8.9 × 10^2^^b^	1060^a^
3	5.4 × 10^4^	3.1 × 10^6^	617^c^
5	1.6 × 10^4^	3.7 × 10^5^	290^d^

a From ref. 14 In the same work redox potentials (at pH > 12) for 1˙/1^−^ and 2˙/2^−^ are 790 mV, and 1000 mV, respectively.^14^

b This is the upper value, the lower *k*^ET^ can be 35 M^−1^ s^−1^, see explanation in the text.

c Measured in water with Britten–Robinson buffer *vs.* Hg|Hg_2_SO_4_|K_2_SO_4_ electrode and recalculated into NHE.^17^

d From ref. 18. This value is very close to *E*^0^ for quercetin at pH 7.^19^

Values of *k*^ET^ listed in second column of Table 2 were calculated assuming that ET is eliminated (*k*^S^ ≈ *k*^HAT^) for reactions carried out in methanol containing 1 M AcOH. Obtained values *k*^ET^ for 1^−^, 3^−^, and 5^−^ (anions) are in reasonable agreement with 1 × 10^4^ M^−1^ s^−1^ calculated by Foti *et al.* (for QH^−^/dpph˙ in methanol)^4*b*^ and with 3.5 × 10^6^ M^−1^ s^−1^ for ET from electron rich anion of 4-methoxyphenol derivative to dpph˙ in acetonitrile.^4*c*^Table 2 presents also the reduction potentials for studied phenols taken from literature (*E*^0^ for dpph˙/dpph^−^,H^+^ in water at pH 7 is 545 mV).^20^*E*^0^ for 5 is 290 mV^18^ making this flavonoid stronger reducing agent than quercetin (*E*^0(pH 7)^ = 330 mV),^19*b*^ that could be reasoned also as an effect of greater participation of 5^−^ than QH^−^ in ET at pH 7 (5 is stronger acid than quercetin). The presented data do not allow to obtain a nice linear correlation of *k*^ET^*versus E*^0^, perhaps, because of large error of estimation of *k*^ET^ values. For electron deficient 2, the upper value of *k*^ET^ was estimated assuming that *k*^HAT^ for 2 and for 1 are the same (that could be reasoned also due to similar values of *E*^0^, see Table 2), however, basing on eqn (5),¶*k*^HAT^ for 2 should be *ca.* 25 times smaller than for 1, thus, *k*^ET^ for 2^−^ might be as low as 35 M^−1^ s^−1^. Such a small *k*^ET^ is not surprising because for 2^−^ this process is the most endergonic among all five ArO^−^/dpph˙ pairs.

The presented *k*^ET^ values are not strictly quantitative and include propagation of experimental errors (especially for 2) but they allow to estimate the concentration-dependent contribution of HAT and ET mechanisms to the overall rate of reaction, see Fig. S41–S43† and graphical abstract. Reaction of dpph˙ with the most acidic and most reactive 5 is dominated by ET within the whole concentration range (Fig. 2 and S43†) with the reaction order close to 0.5 in [5] as a consequence of the Ostwald law of dilution. For 1 (the weakest acid) HAT dominates over ET, whereas for 2 there is an inversion of dominating mechanism at [2] ≈ 50 mM.

## Conclusions

There are many well-documented shortcomings of the dpph˙ assay, including poor or no correlation with the antioxidant activity of phenols measured under physiologically relevant conditions (reactivity toward peroxyl radicals).^5^ Another problem with the dpph˙ assay is that IC_50_ parameter reflects the position of redox equilibrium between dpph˙ and the tested compound, established after the incubation time, and gives no information about the kinetics and stoichiometry of the reaction.^5*b*,*d*^

Kinetic measurements (stopped flow technique, initial rates of reaction) can provide information on the structure-reactivity relationship of putative antioxidants reacting *via* HAT in non-polar solvents,^6^ However, in polar solvents much faster electron transfer is competitive or dominating mechanism and we demonstrated that contribution of HAT/ET to the overall rate is strongly dependent on the concentration of a tested phenol. A careful interpretation of both, kinetic and quasi-kinetic (IC_50_) results, has to be always performed, and mixed HAT/ET mechanism of reaction 1 might produce false results not only in the kinetic experiments but also in IC_50_ assay, as IC_50_ is a parameter strongly related to the concentration of tested phenols (and even expressed in phenol concentration units !). IC_50_ parameter is frequently used for comparison of “antioxidant properties” of phenols and other phytochemicals but our findings indicate a serious limitation of such methodology.

## General experimental procedures

Commercially available phenols 1, 2, and 5 were of the highest purity and were used as received. Macrocyclic polyphenols: *C*-methylcalix[4]resorcinarene (3) and *C*-undecylcalix[4]resorcinarene (4) were prepared following the method proposed by Weinelt and Schneider^21^ by condensation of resorcinol and appropriate aldehyde in ethanol containing aqueous HCl as in our previous work.^12^^1^H NMR of C-alkyl[4]resorcinarenes were recorded on a Varian spectrometer at 300 MHz and 298 K and were compared with literature.^22^3 was obtained by condensation of resorcinol with ethanal. 0.5 mol of resorcinol dissolved in 500 mL of ethanol/water (1/1, v/v) was immersed in an ice bath, then 125 mL of concentrated hydrochloric acid was added. Subsequently, 0.5 mol of ethanal was added dropwise. Then, the reaction mixture was kept at rt and the reaction was carried out for 96 hours with continuous stirring under nitrogen. The mixture was cooled and concentrated under reduced pressure. The obtained precipitate was washed several times with cold ethanol, crystallized form ethanol/water (1 : 1), and dried (yield 40–50%) ^1^H NMR (300 MHz, acetone-*d*_6_) *δ* 8.48 (s, 8H), 7.64 (s, 4H), 6.21 (s, 4H), 4.52 (q, *J* = 7.3 Hz, 4H), 1.76 (d, *J* = 7.4 Hz, 12H), see Fig. S1.†4 was obtained by condensation of resorcinol (0.69 mol) with dodecanal (0.69 mol). The compounds were dissolved in 690 mL of ethanol and cooled in the ice bath to temperature close to 0 °C. Subsequently, 111 mL of concentrated hydrochloric acid was added dropwise to the mixture. Then, the temperature was increased to 70 °C and kept for 12 hours with continuous stirring and nitrogen flow. The mixture was cooled and concentrated under reduced pressure. The obtained precipitate was washed several times with cold ethanol, crystallized from methanol, and dried (yield 20–30%). ^1^H NMR (300 MHz, CDCl_3_) *δ* 9.56 (t, *J* = 20.0 Hz, 4H), 9.28 (t, *J* = 25.0 Hz, 4H), 7.20 (s, 4H), 6.11 (s, 4H), 4.29 (t, *J* = 7.7 Hz, 4H), 2.21 (s, 8H), 1.27 (s, 72H), 0.88 (t, 12H), see Fig. S2.†

Since rate of ArOH/dpph˙ reaction in neat methanol is highly sensitive to traces of acids and bases, prior to the use, methanol was fractionally distilled over a small amount of dpph˙ and a few beads of an acidic ion-exchange resin. Measurements were made following the procedure described previously.^3*c*,23^ Decays of dpph˙ (*ε* ∼ 11 000 M^−1^ cm^−1^) were monitored 517 nm on an Applied Photophysics SX 20 stopped flow spectrometer, equipped with a xenon arc lamp source and photodiode array detector. The mixing cell (10 mm optical path length, dead-time of mixing 1.1 ms) and the tubes delivering the reactants were thermostated at temperature 25 °C. Initial concentrations of dpph˙ were 6–12 × 10^−5^ M for reactions with 1 and 2 and 1–15 × 10^−5^ M for reactions with much more reactive 3–5, *i.e.*, always in the presence of a stoichiometric excess of ArOH. Measurements were made in neat and acidified methanol (with 10, 100 and 1000 mM AcOH) and in mixed 1 : 1 (v/v) methanol–water with pH adjusted to 5.4 (acetate buffer) and pH 7.4 (phosphate buffer). In all experiments, the initial rates (usually determined for 5–10% of dpph˙ conversion) were taken for calculations of *k*_exp_; for example, the conversion of dpph˙ 0.5 s after mixing was 1%, 0.38%, 17%, 29% and 31% for 1–5, respectively, and after 2 seconds the conversion was 3.2%, 0.8%, 50% and 71% for compounds 1–4. The pseudo-first-order rate constants, *k*_exp_, were calculated as average values from at least two independent sets of measurements. Values of bimolecular rate constants, *k*^S^, were calculated as a slope of the straight-line eqn (2). EPR measurements by Staško *et al.*^24^ for mixed ethanol/water systems indicated that dpph˙ behaves as typical solute for a lower water ratio of 0–60% (v/v) but at a higher water content (above 60%) dpph˙ forms microaggregates (still without precipitation). We assume, therefore, that dpph˙ forms a homogeneous system with water/methanol (1 : 1, v/v) during our experiments at pH 5.4 and 7.4.

## Author contributions

Conceptualization (GL and PP), funding acquisition (GL), investigation (PP, AK, PŁ), methodology, supervision and project administration (GL), visualization (KJP and GL), writing (all authors).

## Conflicts of interest

There are no conflicts to declare.

## Supplementary Material

RA-012-D2RA01033J-s001
