# Supplementary material for: Concentration-dependent HAT/ET mechanism of the reaction of phenols with 2,2-diphenyl-1-picrylhydrazyl (dpph˙) in methanol
Source: RSC Adv. 2022 Mar 15;12(13):8131–6. doi: 10.1039/d2ra01033j (PMC8982332; doi:10.1039/d2ra01033j)
Supplement: RA-012-D2RA01033J-s001 [file RA-012-D2RA01033J-s001.pdf]

Electronic Supplementary Material (ESI) for RSC Advances.
This journal is © The Royal Society of Chemistry 2022

Electronic Supplementary Information

Concentration dependent HAT/ET mechanism of reaction of phenols with 2,2-diphenyl-1-picrylhydrazyl (dpph•) in methanol

Paweł Przybylski,[a] Adrian Konopko,[a,b] Piotr Łętowski,[a]
Katarzyna Jodko-Piórecka,[a] & Grzegorz Litwinienko[a*]

[a] University of Warsaw, Faculty of Chemistry, Pasteura 1, 02-093 Warsaw, Poland.
[b] Instytut of Experimental Biology, Polish Academy of Sciences, Pasteura 3, 02-093 Warsaw, Poland.

SYMBOLS and ABBREVIATIONS

**1**	phenol
**2**	4-OH-acetophenone
**3**	C-methylcalix[4]resorcinarene
**4**	C-undecylcalix[4]resorcinarene
**5**	baicalein
AcOH	acetic acid
MeOH	methanol

TABLE OF CONTENTS

	Title	page
	Experimental procedures	S3
**Figure S1.**	^{1}H NMR spectrum of **3**.	S-4
**Figure S2.**	^{1}H NMR spectrum of **4**.	S-4
**Figure S3**.	Examples of kinetic traces for dpph• bleaching after mixing with **1**.	S-5
**Figure S4**.	Examples of kinetic traces for dpph• bleaching after mixing with **2**.	S-5
**Figure S5**.	Examples of kinetic traces for dpph• bleaching after mixing with: **3** and **4**.	S-6
**Figure S6**	Examples of kinetic traces for dpph• bleaching after mixing with **5**.	S-6
**Table S1.** **Figure S7.** **Figure S8.**	Data and kinetic parameters for reaction of dpph• with excess of **1** in MeOH, and with linear and nonlinear fitting.	S-7
**Table S2.** **Figure S9.**	Data and kinetic parameters for reaction of dpph• with excess of **1** in acidified MeOH (10mM AcOH) and linear fitting.	S-8
**Table S3.** **Figure S10.**	Data and kinetic parameters for reaction of dpph• with excess of **1** in acidified MeOH (100mM AcOH) and linear fitting.	S-9
**Table S4.** **Figure S11.**	Data and kinetic parameters for reaction of dpph• with excess of **1**in acidified MeOH (1000mM AcOH) and linear fitting.	S-10
**Table S5.** **Figure S12.** **Figure S13.**	Data and kinetic parameters for reaction of dpph• with excess of **2** in MeOH, linear and nonlinear fitting.	S-11
**Table S6.** **Figure S14.** **Figure S15.**	Data and kinetic parameters for reaction of dpph• with excess of **2** in acidified MeOH (10mM AcOH), linear and nonlinear fitting.	S-13
**Table S7.** **Figure S16.** **Figure S17.**	Data and kinetic parameters for reaction of dpph• with excess of **2** in acidified MeOH (100mM AcOH), linear and nonlinear fitting.	S-15

**Table S8. Figure S18. Figure S19.**	Data and kinetic parameters for reaction of dpph• with excess of **2** in acidified MeOH (1M AcOH), linear and nonlinear fitting.	S-17
**Table S9. Figure S20.**	Data and kinetic parameters for reaction of dpph• with excess of **1** in 50% MeOH at pH = 5.4, and linear fitting.	S-19
**Table S10. Figure S21.**	Data and kinetic parameters for reaction of dpph• with excess of **1** in 50% MeOH at pH = 7.4, and linear fitting.	S-20
**Table S11. Figure S22.**	Data and kinetic parameters for reaction of dpph• with excess of **2** in 50% MeOH at pH = 5.4, and linear fitting.	S-21
**Table S12. Figure S23.**	Data and kinetic parameters for reaction of dpph• with excess of **2** in 50% MeOH at pH = 7.4, and linear fitting.	S-22
**Table S13. Figure S24. Figure S25.**	Data and kinetic parameters for reaction of dpph• with excess of **3** in MeOH, linear and nonlinear fitting.	S-23
**Table S14. Figure S26.**	Data and kinetic parameters for reaction of dpph• with excess of **3** in acidified MeOH (10mM AcOH) and linear fitting.	S-25
**Table S15. Figure S27.**	Data and kinetic parameters for reaction of dpph• with excess of **3** in acidified MeOH (100mM AcOH) and linear fitting.	S-26
**Table S16. Figure S28.**	Data and kinetic parameters for reaction of dpph• with excess of **3** in acidified MeOH (1000mM AcOH) and linear fitting.	S-27
**Table S17. Figure S29. Figure S30.**	Data and kinetic parameters for reaction of dpph• with excess of **4** in MeOH, linear and nonlinear fitting.	S-28
**Table S18. Figure S31.**	Data and kinetic parameters for reaction of dpph• with excess of **4** in acidified MeOH (10mM AcOH) and linear fitting.	S-30
**Table S19. Figure S32.**	Data and kinetic parameters for reaction of dpph• with excess of **4** in acidified MeOH (100mM AcOH) and linear fitting.	S-31
**Table S20. Figure S33.**	Data and kinetic parameters for reaction of dpph• with excess of **4** in acidified MeOH (1000mM AcOH) and linear fitting.	S-32
**Table S21. Figure S34. Figure S35.**	Data and kinetic parameters for reaction of dpph• with excess of **5** in MeOH, linear and nonlinear fitting.	S-33
**Table S22. Figure S36.**	Data and kinetic parameters for reaction of dpph• with excess of **5** in acidified MeOH (10mM AcOH) and linear fitting.	S-35
**Table S23. Figure S37.**	Data and kinetic parameters for reaction of dpph• with excess of **5** in acidified MeOH (100mM AcOH) and linear fitting.	S-36
**Table S24. Figure S38.**	Data and kinetic parameters for reaction of dpph• with excess of **5** in acidified MeOH (1000mM AcOH) and linear fitting.	S-37
**Figure S39.**	Dissociation diagrams for **1** and **2**.	S-38
**Figure S40.**	Dissociation diagrams for **3** and **5**.	S-39
	Calculation of the rate constants for electron transfer from phenolate anions to dpph$^{\bullet}$, with comments.	S-40
**Table S25.**	Parameters used for estimation of k^{ET}.	S-40
**Figure S41.**	Fraction of anion[ArO$^-$] plotted versus [ArOH] and contribution of HAT /ET to the overall rate of reaction of **2** (and **2**$^-$) with dpph$^{\bullet}$ in methanol.	S-41
**Figure S42.**	Fraction of anion[ArO$^-$] plotted versus [ArOH] and contribution of HAT /ET to the overall rate of reaction of **3** (and **3**$^-$) with dpph$^{\bullet}$ in methanol.	S-41
**Figure S43.**	Fraction of anion[ArO$^-$] plotted versus [ArOH] and contribution of HAT /ET to the overall rate of reaction of **5** (and **5**$^-$) with dpph$^{\bullet}$ in methanol.	S-41
	REFERENCES	S-42

Experimental

Commercially available phenols **1**, **2** and **5** were of the highest purity and were used as received. Macrocyclic polyphenols: C-methylcalix[4]resorcinarene (**3**) and C-undecylcalix[4]resorcinarene (**4**) were prepared following the method proposed by Weinelt and Schneider[1] by condensation of resorcinol and appropriate aldehyde in ethanol containing aqueous HCl as in our previous work.[2] ^{1}H NMR of C-alkyl[4]resorcinarenes were recorded on a Varian spectrometer at 300 MHz and 298K and were compared with literature.[3]

3 was obtained by condensation of resorcinol with ethanal. 0.5 mol of resorcinol dissolved in 500 mL of ethanol /water (1/1, v/v) was immersed in an ice bath, then 125 mL of concentrated hydrochloric acid was added. After that, 0.5 mol of ethanal was added dropwise. Then, temperature was allowed to cool down to rt, and the reaction was carried out during 96 hours with continuous stirring under nitrogen. The mixture was cooled and concentrated under reduced pressure. The obtained precipitate was washed several times with cold ethanol, crystallized form ethanol/ water (1:1), and dried (yield 40-50%). ^{1}H NMR (300 MHz, Acetone-d_6) δ 8.48 (s, 8H), 7.64 (s, 4H), 6.21 (s, 4H), 4.52 (q, J = 7.3 Hz, 4H), 1.76 (d, J = 7.4 Hz, 12H). Signals at 2.95 ppm comes from water[2, 3b, 4] and 2.09 ppm from traces of aldehyde (substrate), see Fig. S1.

4 was obtained by condensation of resorcinol (0.69 mol) with dodecanal (0.69 mol). Compounds were dissolved in 690 mL of ethanol and cooled in the ice bath to temperature close to 0 °C. After that 111 mL of concentrated hydrochloric acid was added dropwise to the mixture. Then, the temperature was increased to 70 °C, and kept during 12 hours with continuous stirring and nitrogen flow. The mixture was cooled and concentrated under reduced pressure. The obtained precipitate was washed several times with cold ethanol, crystallized from methanol and dried (yield 20-30%). ^{1}H NMR (300 MHz, $CDCl_3$) δ 9.56 (t, J = 20.0 Hz, 4H), 9.28 (t, J = 25.0 Hz, 4H), 7.20 (s, 4H), 6.11 (s, 4H), 4.29 (t, J = 7.7 Hz, 4H), 2.21 (s, 8H), 1.27 (s, 72H), 0.88 (t, 12H). Signal at 2.95 ppm is from traces of persistently present water, in agreement with several reports[2, 3b, 4], see Fig. S2.

Since rate of ArOH/dpph$^\bullet$ reaction in neat methanol is highly sensitive to traces of acids and bases, prior to the use methanol was fractionally distilled over a small amount of dpph$^\bullet$ and a few beads of an acidic ion-exchange resin. The measurements were made following the procedure described previously.[5] Decays of dpph$^\bullet$ (ε ~ 11 000 $M^{-1}cm^{-1}$, initial concentrations 6-12 × 10^{-5} M for 1 and 2 and 1-15 × 10^{-5} M for much more reactive **3-5** in the presence of a stoichiometric excess of the phenol were monitored 517 nm on an Applied Photophysics SX 20 stopped flow spectrometer, equipped with a xenon arc lamp source and photodiode array detector. Mixing cell (10 mm optical path length, dead-time of mixing 1.1 ms) and the tubes delivering the reactants were thermostated at temp. 25°C. Measurements were conducted in neat and acidified methanol (with 10, 100, and 1000 mM AcOH) and in mixed 1:1 (v/v) methanol-water with pH adjusted to 5.4 (acetate buffer), and pH 7.4 (phosphate buffer). The initial rates of reaction (usually 5-10% of dpph$^\bullet$ conversion, see footnote 9) were monitored giving the pseudo-first-order rate constants, k_{exp}, calculated as average values from at least two independent sets of measurements. Values of bimolecular rate constants, k^S were calculated as a slope of the straight-line equation 3. EPR measurements by Staško et al.[6] for mixed ethanol/water systems indicated that dpph$^\bullet$ behaves as typical solute for lower water ratio 0-60% (v/v) but at higher water content (above 60%) dpph$^\bullet$ forms microaggregates (still without precipitation). We assume dpph$^\bullet$ forms a homogeneous system with water /methanol (1:1, v/v).

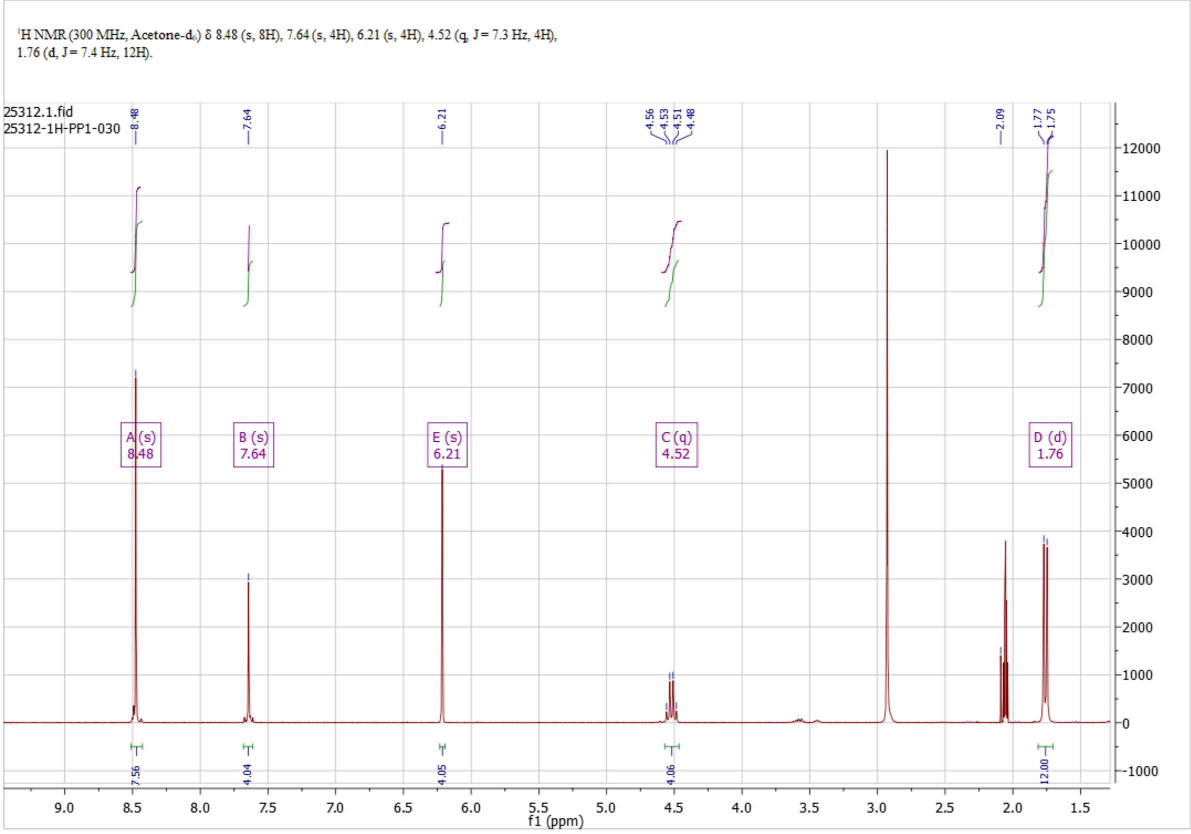

Figure S1. ^{1}H NMR spectrum of **3** recrystallized from ethanol/water (1/1).

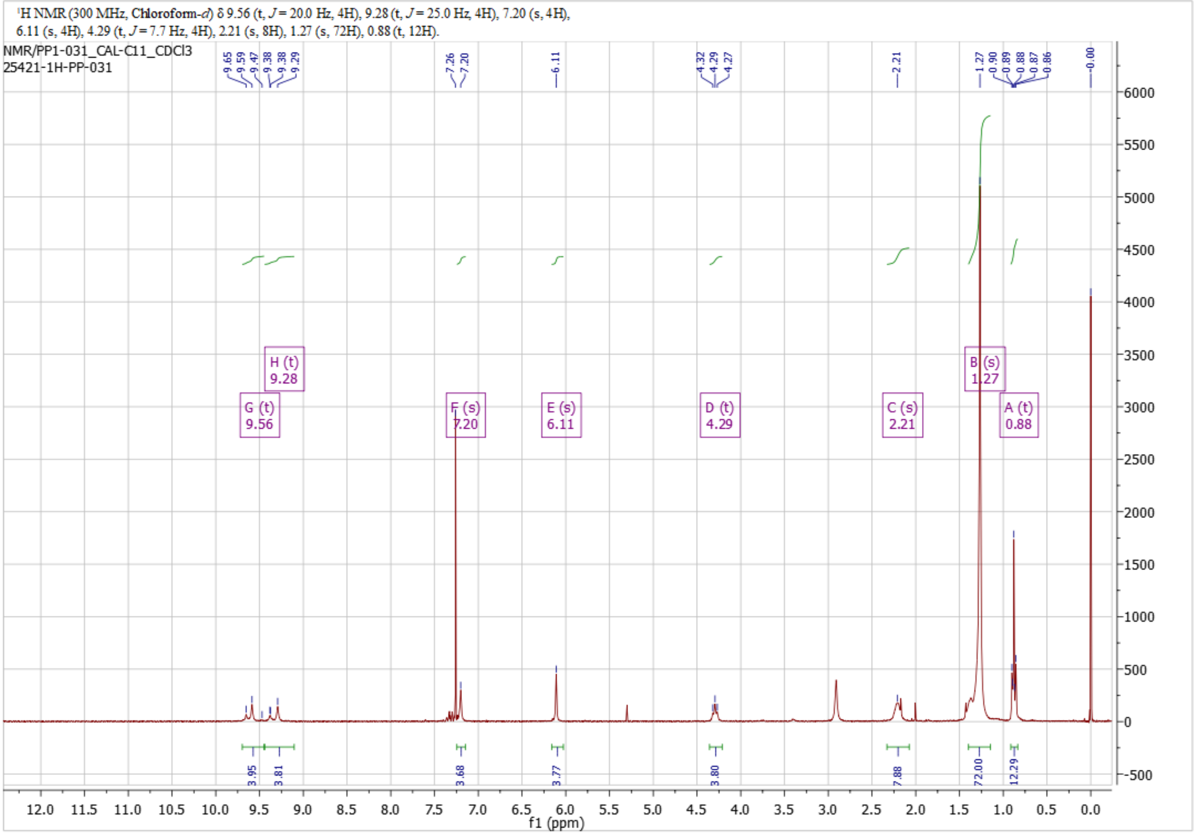

Figure S2. ^{1}H NMR spectrum of **4** recrystallized from methanol

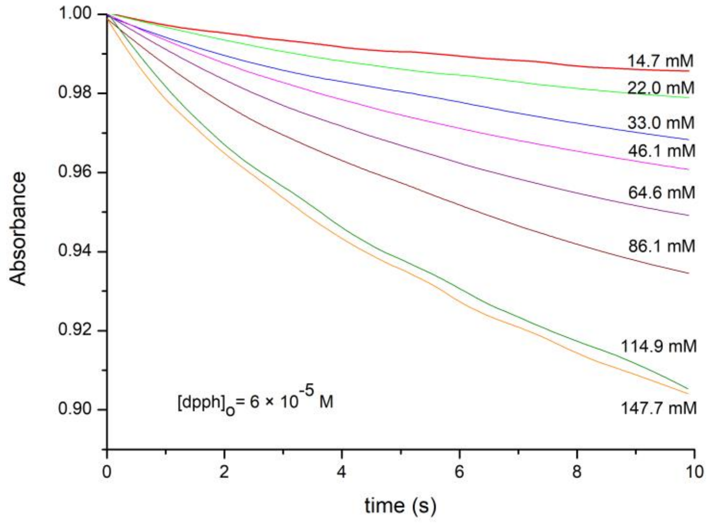

Figure S3. Examples of kinetic traces for dpph$^{\bullet}$ bleaching after mixing with **1** at concentrations 14-150 mM.

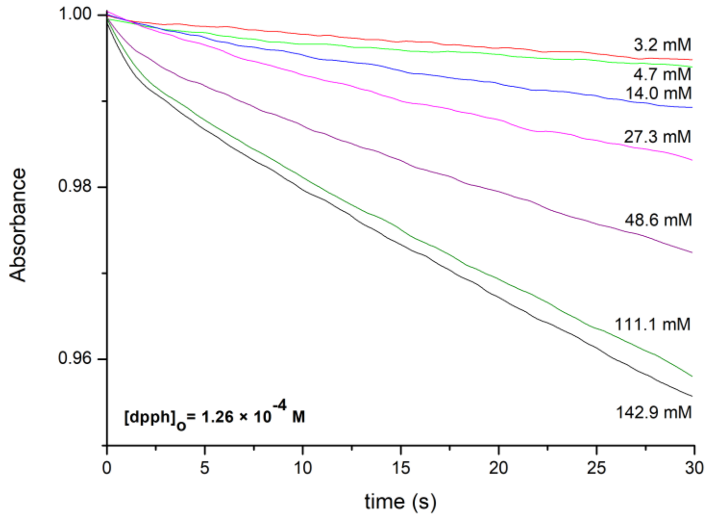

Figure S4. Examples of kinetic traces for dpph$^{\bullet}$ bleaching after mixing with **2** at concentrations 3-143 mM.

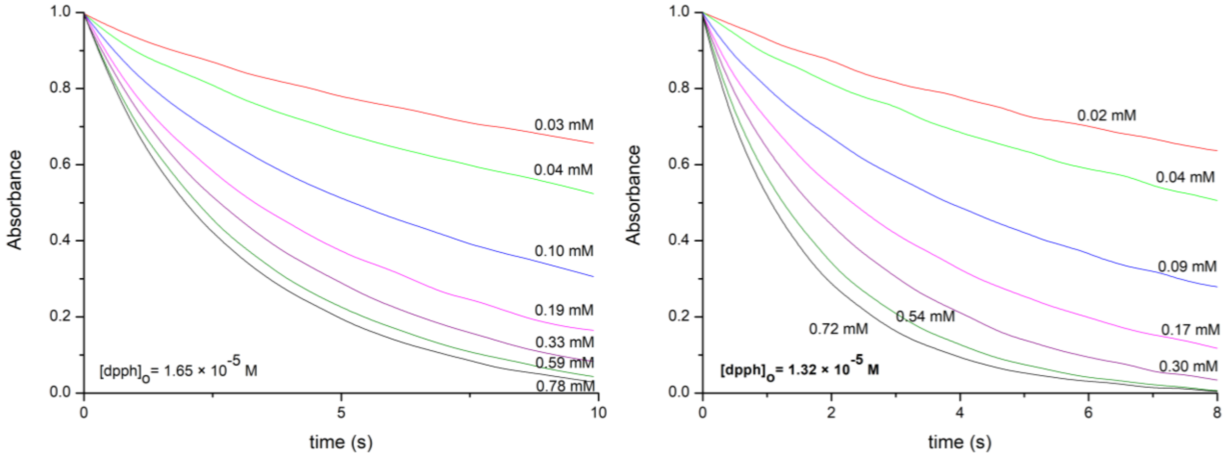

Figure S5. Examples of kinetic traces for dpph$^{\bullet}$ bleaching after mixing with: **3** at concentrations (3 – 80) × 10^{-5} M (left panel), and **4** at concentrations (2 – 75) × 10^{-5} M (right panel).

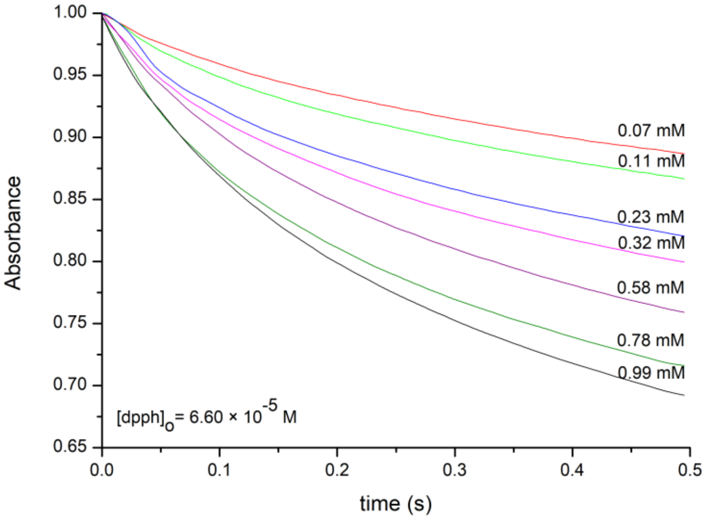

Figure S6. Examples of kinetic traces for dpph$^{\bullet}$ bleaching after mixing with **5** at concentrations (7 – 100) × 10^{-5} M.

Table S1. Pseudo-first-order rate constant (k_{exp}) for reaction of **dpph•** (constant initial concentration) with excess of **1** (PhOH) in MeOH and bimolecular rate constant k^S calculated as a slope from linear dependence of k_{exp} plotted against concentration of phenol: $k_{exp}=k^S[ArOH]$ + const, with regression coefficient (R^2) and error $\Delta k^S_{90\%}$ calculated as standard deviation for confidential level 90%.

Neat MeOH		Neat MeOH	
[**1**] /mM	$k_{ex} \times 10^3$ /s^{-1}	[**1**] /mM	$k_{ex} \times 10^3$ /s^{-1}
147.7	11.3	141.7	11.0
114.9	9.3	110.2	8.8
86.1	7.3	82.6	7.0
64.6	5.9	62.0	5.5
46.1	4.3	44.3	4.2
33.0	3.4	31.6	3.1
22.0	2.2	21.1	2.3
14.7	1.6	14.1	1.7
$k^S = 0.073$ M^{-1}s^{-1}		$k^S = 0.073$ M^{-1}s^{-1}	
$\Delta k^S_{90\%} = 0.004$		$\Delta k^S_{90\%} = 0.002$	
$R^2 = 0.9947$		$R^2 = 0.9984$	

$$\mathbf{k^{MeOH} = (73 \pm 4) \times 10^{-3}\ M^{-1}s^{-1}}$$

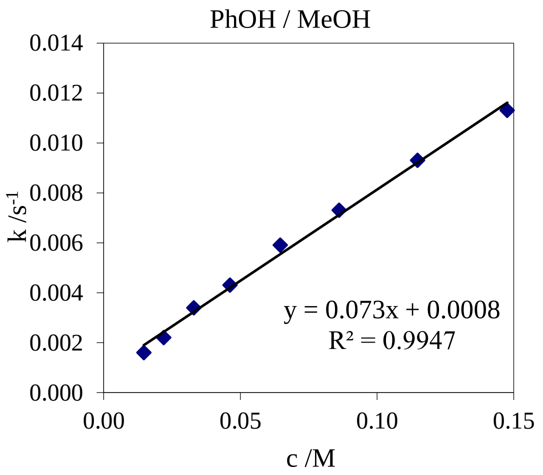

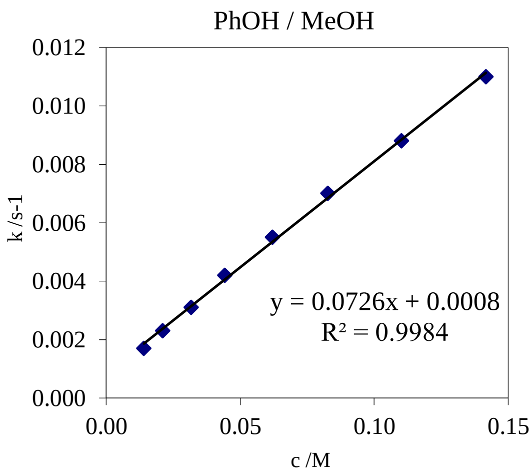

Figure S7. Plots of k_{ex} versus concentration of **1** (data from Table S1).

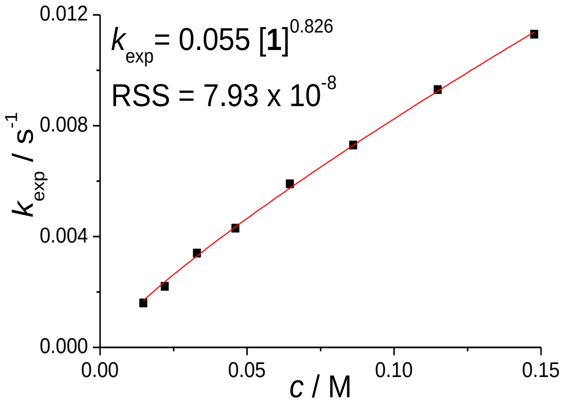

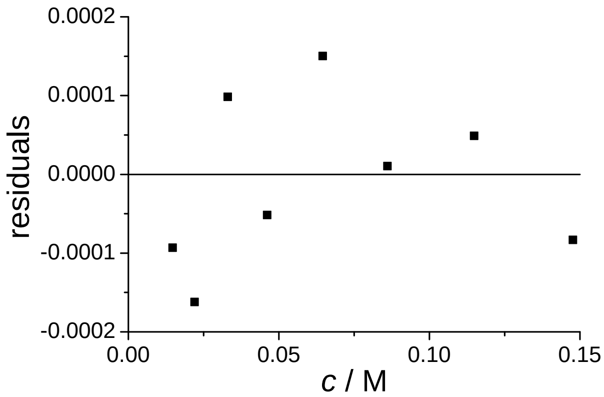

Figure S8. Nonlinear fitting of experimental k_{exp} versus concentration of [**1**] (the set od data from the left column of Table 1) together with residuals of fitting.

Table S2. Kinetic data for the reaction of **dpph•** with **1** in acidified MeOH. Symbols and abbreviations are the same as in caption to Table S1.

**MeOH/ 10mM AcOH**		**MeOH/ 10mM AcOH**	
[**1**] /mM	$k_{ex} \times 10^4$ /s^{-1}	[**1**] / mM	$k_{ex} \times 10^4$ /s^{-1}
141.6	20.0	148.8	20.0
110.2	17.7	115.8	16.0
82.6	15.0	86.8	13.0
62.0	12.0	65.1	11.0
44.3	10.5	46.5	9.0
31.6	9.0	33.2	7.3
21.1	8.0	22.1	6.2
14.1	7.0	14.8	5.0
k^S = 0.010 M^{-1}s^{-1}		k^S = 0.011 M^{-1}s^{-1}	
$\Delta k^S_{90\%}$ = 0.001		$\Delta k^S_{90\%}$ = 0.001	
R^2 = 0.9937		R^2 = 0.9982	

$\mathbf{k^{MeOH/10mM\ AcOH} = (11 \pm 1) \times 10^{-3}\ M^{-1}s^{-1}}$

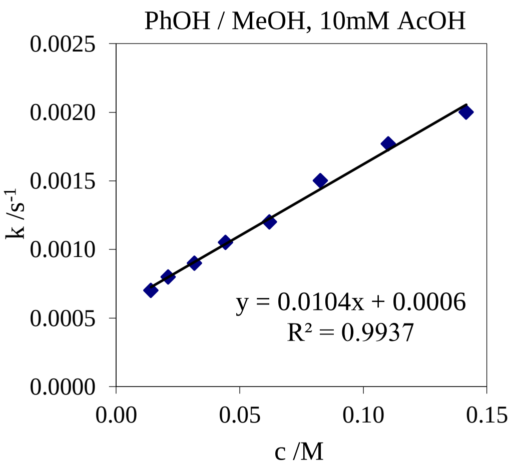

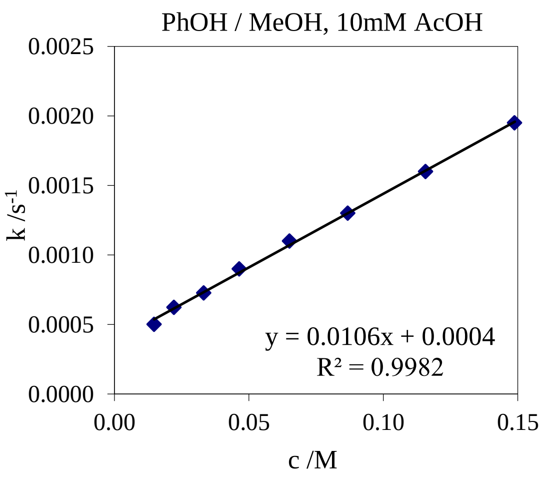

Figure S9. Plots of k_{ex} versus concentration of **1** (data from Table S2).

Table S3. Kinetic data for the reaction of **dpph•** with **1**in MeOH with 100 mM AcOH. Symbols and abbreviations are the same as in caption to Table S1.

**MeOH/ 100mM AcOH**		**MeOH/ 100mM AcOH**	
[**1**] /mM	$k_{exp} \times 10^4$ /s^{-1}	[PhOH] /mM	$k_{exp} \times 10^4$ /s^{-1}
127.6	15.0	147.8	16.0
99.3	13.0	115.0	13.0
74.5	10.0	86.2	11.0
55.8	8.0	64.7	9.0
26.6	5.3	46.2	7.0
17.7	4.8	30.8	6.0
10.6	4.4	20.5	5.0
		12.3	4.0
$k^S = 0.010$ M^{-1}s^{-1}		$k^S = 0.009$ M^{-1}s^{-1}	
$\Delta k^S_{90\%} = 0.001$		$\Delta k^S_{90\%} = 0.001$	
$R^2 = 0.9941$		$R^2 = 0.9976$	

$$\mathbf{k^{MeOH/100\ mMacetic} = (10 \pm 2) \times 10^{-3}\ M^{-1}s^{-1}}$$

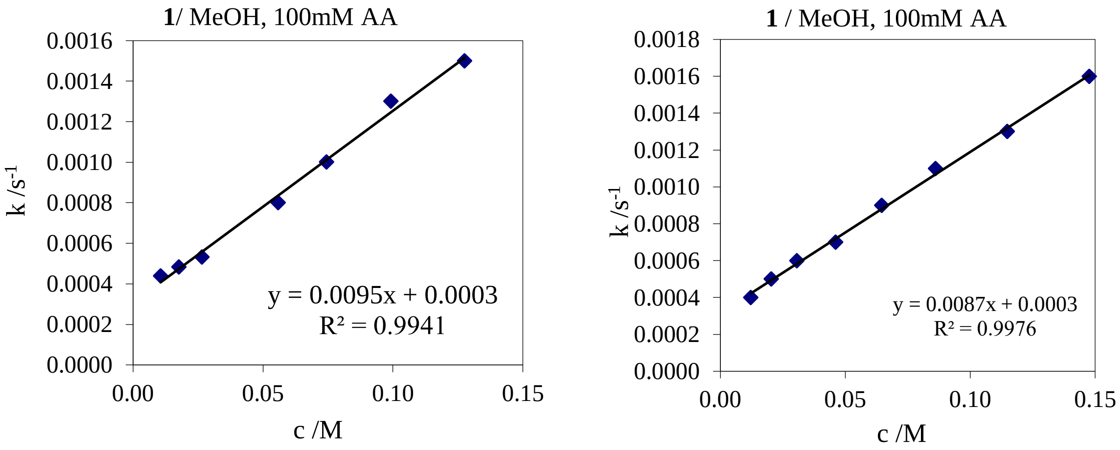

Figure S10. Plots of k_{ex} versus concentration of **1** (data from Table S3).

Table S4. Kinetic data for the reaction of **dpph•** with **1**in MeOH with 1M AcOH. Symbols and abbreviations are the same as in caption to Table S1.

**MeOH/ 1000mM AcOH**		**MeOH/ 1000mM AcOH**	
[**1**] /mM	$k_{ex} \times 10^4$ /s^{-1}	[PhOH] /mM	$k_{ex} \times 10^4$ /s^{-1}
151.1	8.9	158.4	13.0
117.5	7.6	118.8	10.0
88.1	6.3	89.1	8.0
66.1	5.0	66.8	7.0
33.7	3.7	47.7	6.0
22.5	3.3	34.1	5.0
15.0	2.2	22.7	4.0
		15.2	3.0
$k^S = 0.005\ M^{-1}s^{-1}$		$k^S = 0.007\ M^{-1}s^{-1}$	
$\Delta k^S_{90\%} = 0.001$		$\Delta k^S_{90\%} = 0.001$	
$R^2 = 0.9904$		$R^2 = 0.9912$	

$$\boldsymbol{k^{MeOH/1000\ mMAcOH} = (6 \pm 1)x\ 10^{-3}\ M^{-1}s^{-1}}$$

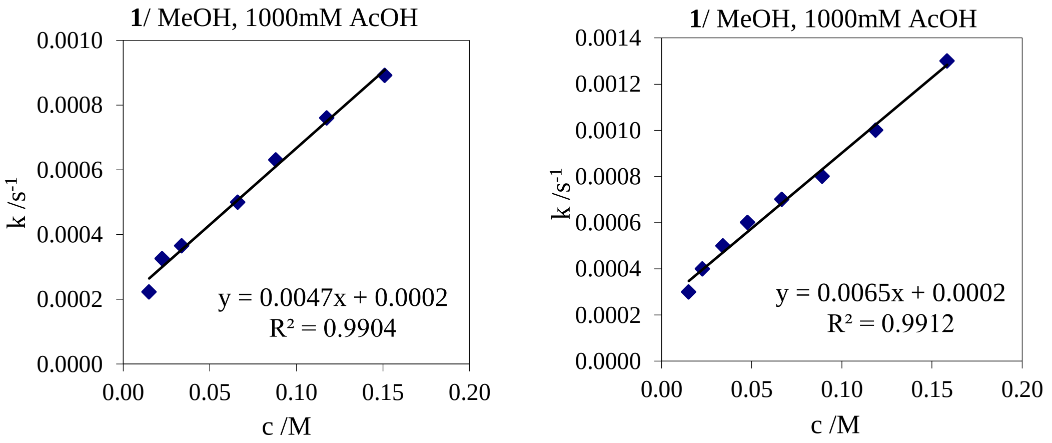

Figure S11. Plots of k_{ex} versus concentration of **1** (data from Table S4).

Summary of parameters determined for 1in methanol:

k^{MeOH} = $(73 \pm 4) \times 10^{-3}\ M^{-1}s^{-1}$

$k^{MeOH/10\ mMAcOH}$ = $(11 \pm 1) \times 10^{-3}\ M^{-1}s^{-1}$

$k^{MeOH/100\ mMAcOH}$ = $(10 \pm 2) \times 10^{-3}\ M^{-1}s^{-1}$

$k^{MeOH/1000\ mMAcOH}$ = $(6 \pm 1) \times 10^{-3}\ M^{-1}s^{-1}$

Table S5. Kinetic data for the reaction of **dpph•** with **2** in MeOH. Symbols and abbreviations are the same as in caption to Table S1.

**Neat MeOH**		**Neat MeOH**	
[**2**] /mM	$k_{exp} \times 10^3$ /s^{-1}	[**2**] /mM	$k_{exp} \times 10^3$ /s^{-1}
183.7	1.5	183.7	1.5
142.9	1.4	142.9	1.4
111.1	1.3	111.1	1.2
86.4	1.2	86.4	1.1
64.8	1	64.8	1
48.6	0.9		
$k^S = 4.4\times10^{-3}$ M^{-1}s^{-1}		$k^S = 4.4\times10^{-3}$ M^{-1}s^{-1}	
$\Delta k^S_{90\%} = 1.1\times10^{-3}$		$\Delta k^S_{90\%} = 0.7\times10^{-3}$	
$R^2 = 0.9339$		$R^2 = 0.9795$	
48.6	0.9	64.8	1.0
36.5	0.7	48.6	0.8
27.3	0.6	36.5	0.7
19.5	0.5	27.3	0.6
14.0	0.4	19.5	0.4
10.0	0.3	14.0	0.3
7.1	0.3	10.0	0.3
4.7	0.2	7.1	0.2
3.2	0.1	4.7	0.2
2.1	0.08	3.2	0.1
1.4	0.07	2.1	0.09
		1.4	0.06
$k^S = 17.2\times10^{-3}$ M^{-1}s^{-1}		$k^S = 14.9\times10^{-3}$ M^{-1}s^{-1}	
$\Delta k^S_{90\%} = 2.8\times10^{-3}$		$\Delta k^S_{90\%} = 1.4\times10^{-3}$	
$R^2 = 0.9643$		$R^2 = 0.9733$	

$$\boldsymbol{k^{MeOH} = (4.4 \pm 0.7) \times 10^{-3}\ M^{-1}s^{-1}}$$
$$\boldsymbol{k^{MeOH} = (16.1 \pm 2.8) \times 10^{-3}\ M^{-1}s^{-1}}$$

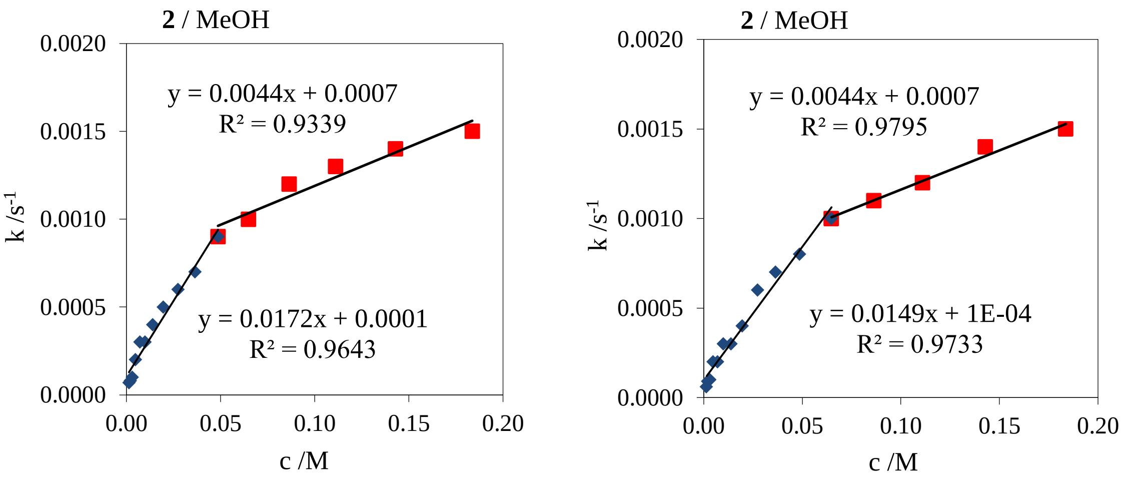

Figure S12. Linear fitting for k_{ex} versus concentration of **2** (data from Table S5).

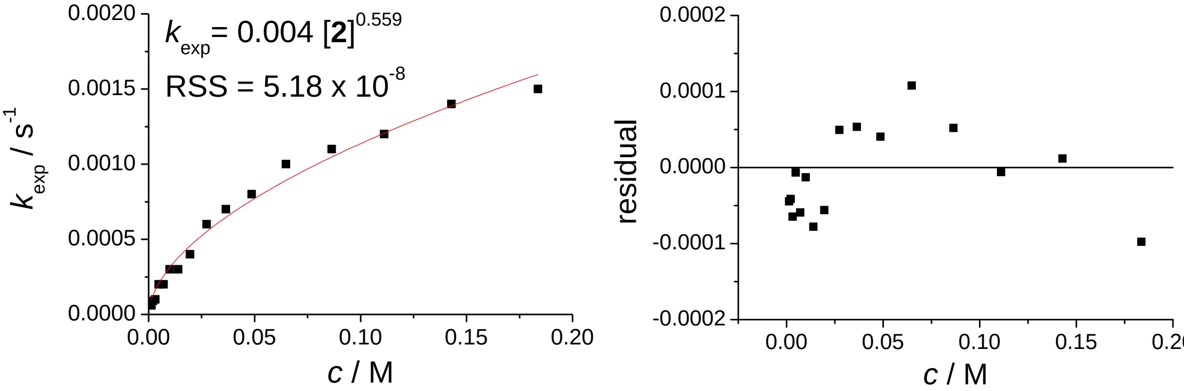

Figure S13. Nonlinear fitting of experimental k_{exp} versus concentration of [**2**] (the set of experimental data from the right column of Table S5) together with residuals of fitting.

Table S6. Kinetic data for the reaction of **dpph•** with **2** in MeOH with AcOH (10 mM). Symbols and abbreviations are the same as in caption to Table S1.

**MeOH/ 10mM AcOH**		**MeOH/ 10mM AcOH**	
[**2**] /mM	$k_{exp} \times 10^3$ /s^{-1}	[**2**] /mM	$k_{exp} \times 10^3$ /s^{-1}
183.6	1.2	183.6	1.2
142.8	1.0	142.8	1.1
111.1	1.0	111.1	1.0
83.3	0.8	83.3	0.8
62.5	0.7	62.5	0.7
$k^S = 3.9\times10^{-3}$ $M^{-1}s^{-1}$		$k^S = 4.2\times10^{-3}$ $M^{-1}s^{-1}$	
$\Delta k^S_{90\%} = 1.2\times10^{-3}$		$\Delta k^S_{90\%} = 1.1\times10^{-3}$	
$R^2 = 0.9384$		$R^2 = 0.953$	
62.5	0.7	62.5	0.7
46.9	0.6	46.9	0.5
33.5	0.4	33.5	0.3
23.9	0.3	23.9	0.2
17.1	0.2	17.1	0.2
11.4	0.2	11.4	0.1
7.6	0.1	7.6	0.05
5.1	0.1	5.1	0.05
$k^S = 11.1\times10^{-3}$ $M^{-1}s^{-1}$		$k^S = 11.3\times10^{-3}$ $M^{-1}s^{-1}$	
$\Delta k^S_{90\%} = 1.0\times10^{-3}$		$\Delta k^S_{90\%} = 1.1\times10^{-3}$	
$R^2 = 0.9852$		$R^2 = 0.9828$	

$$\boldsymbol{k^{\mathrm{MeOH/10mM\ AcOH}} = (4.0 \pm 1.2)\times10^{-3}\ M^{-1}s^{-1}}$$

$$\boldsymbol{k^{\mathrm{MeOH/10mM\ AcOH}} = (11.2 \pm 0.1)\times10^{-3}\ M^{-1}s^{-1}}$$

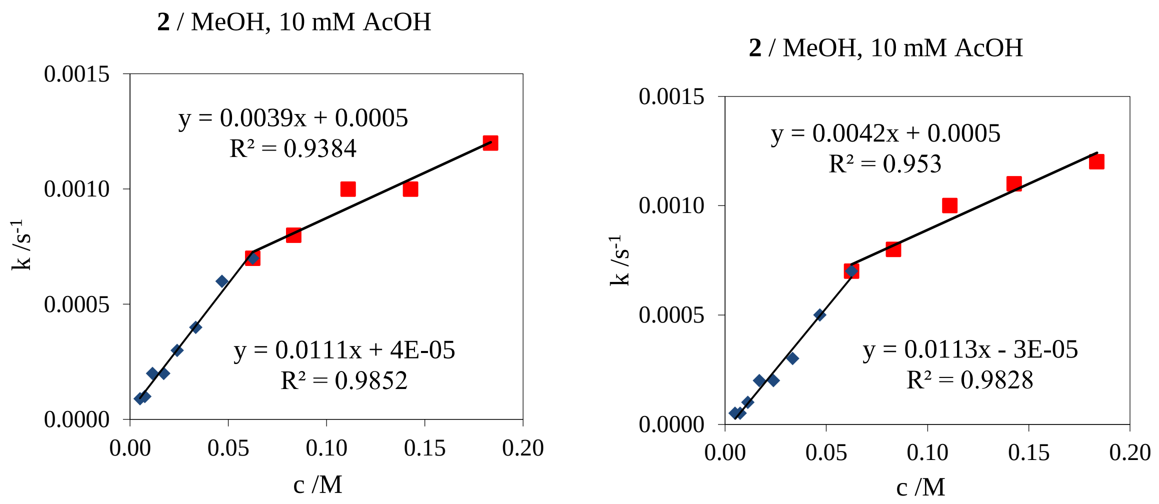

Figure S14. Plots of k_{ex} versus concentration of **2** (data from Table S6).

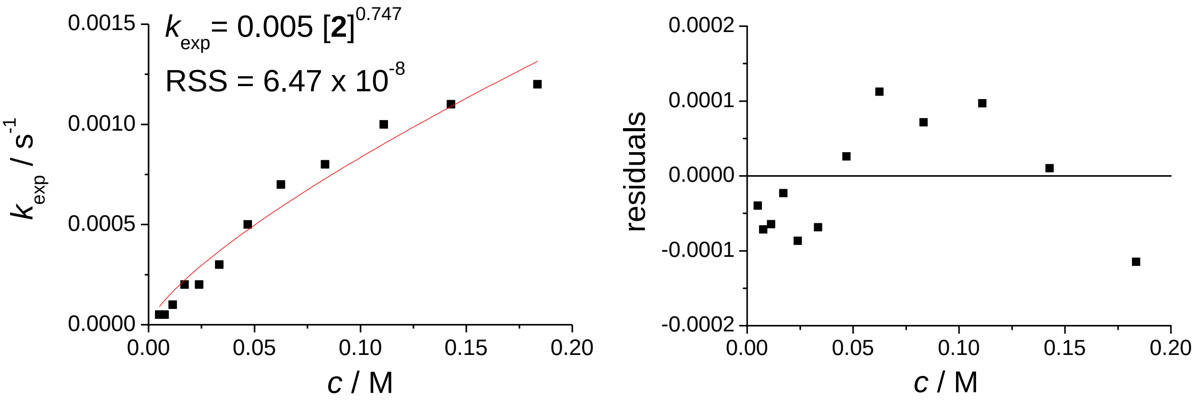

Figure S15. Nonlinear fitting of experimental k_{exp} versus concentration of [**2**] in methanol /10 mM acetic acid (the set of experimental data from the right column of Table S6) together with residuals of fitting.

Table S7. Kinetic data for the reaction of **dpph•** with **2** in MeOH with AcOH (100 mM). Symbols and abbreviations are the same as in caption to Table S1.

**MeOH/ 100mM AcOH**		**MeOH/ 100mM AcOH**	
[**2**] /mM	$k_{exp} \times 10^3$ /s^{-1}	[**2**] /mM	$k_{exp} \times 10^3$ /s^{-1}
183.7	1.1	183.6	1.6
142.9	1.0	142.8	1.4
111.1	1.0	111.1	1.1
83.3	0.9	83.3	1.0
62.5	0.8	62.5	0.8
$k^S = 2.3\times10^{-3}$ M^{-1}s^{-1}		$k^S = 6.6\times10^{-3}$ M^{-1}s^{-1}	
$\Delta k^S_{90\%} = 0.8\times10^{-3}$		$\Delta k^S_{90\%} = 1.0\times10^{-3}$	
$R^2 = 0.9056$		$R^2 = 0.9836$	
62.5	0.8	62.5	0.8
46.9	0.6	46.9	0.7
33.5	0.5	33.5	0.5
23.9	0.4	23.9	0.5
17.1	0.3	17.1	0.3
11.4	0.2	11.4	0.3
7.6	0.2	7.6	0.2
5.1	0.1	5.1	0.1
$k^S = 11.5\times10^{-3}$ M^{-1}s^{-1}		$k^S = 11.6\times10^{-3}$ M^{-1}s^{-1}	
$\Delta k^S_{90\%} = 1.1\times10^{-3}$		$\Delta k^S_{90\%} = 2.1\times10^{-3}$	
$R^2 = 0.984$		$R^2 = 0.9453$	

$$k^{\mathrm{MeOH/100mM\ AcOH}} = (4.5 \pm 2.2)\times10^{-3}\ \mathrm{M^{-1}s^{-1}}$$

$$k^{\mathrm{MeOH/100mM\ AcOH}} = (11.6 \pm 2.1)\times10^{-3}\ \mathrm{M^{-1}s^{-1}}$$

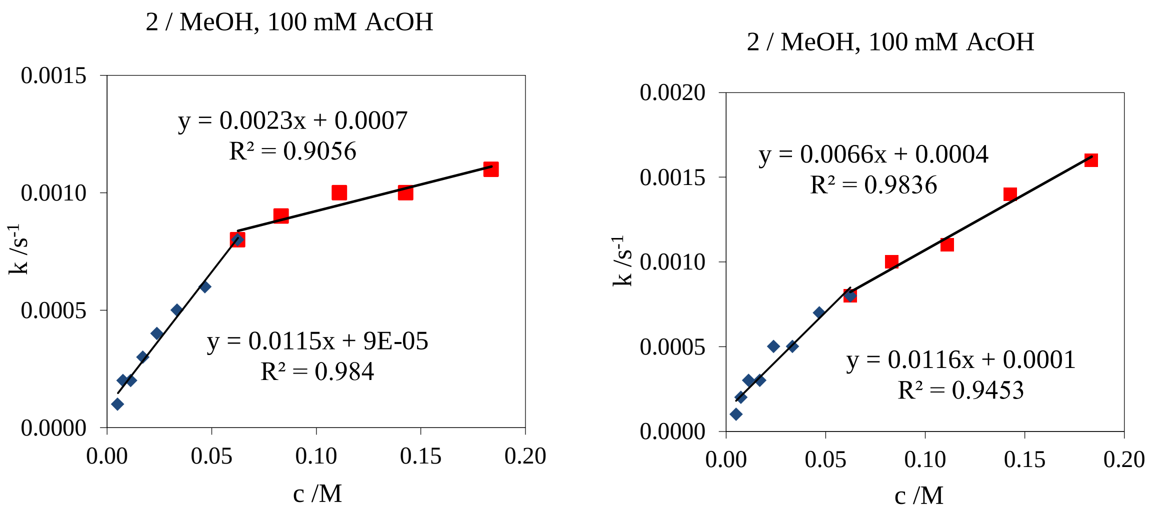

Figure S16. Linear fitting for k_{ex} versus concentration of **2** in methanol /100 mM AcOH (data from Table S7).

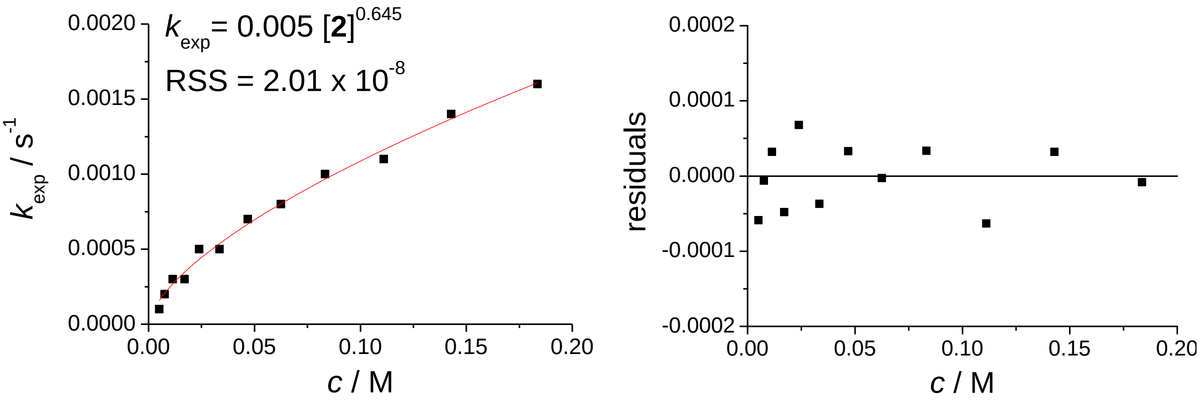

Figure S17. Nonlinear fitting of experimental k_{exp} versus concentration of [**2**] in methanol /100 mM acetic acid (the set of experimental data from the right column of Table S7) together with residuals of fitting.

Table S8. Kinetic data for the reaction of **dpph•** with **2** in MeOH with AcOH (1M). Symbols and abbreviations are the same as in caption to Table S1.

**MeOH/ 1000mM AcOH**		**MeOH/ 1000mM AcOH**	
[**2**] /mM	$k_{exp} \times 10^3$ /s^{-1}	[**2**] /mM	$k_{exp} \times 10^3$ /s^{-1}
183.9	1.1	183.7	1.9
143.0	1.1	142.9	1.6
111.3	1.1	111.1	1.3
83.4	1.0	83.3	1.1
62.6	1.0	62.5	0.9
46.9	0.9		
$k^S = 1.4 \times 10^{-3}$ $M^{-1}s^{-1}$		$k^S = 8.2 \times 10^{-3}$ $M^{-1}s^{-1}$	
$\Delta k^S_{90\%} = 0.8 \times 10^{-3}$		$\Delta k^S_{90\%} = 0.3 \times 10^{-3}$	
$R^2 = 0.7353$		$R^2 = 0.9983$	
46.9	0.9	62.5	0.9
33.5	0.7	46.9	0.8
23.9	0.5	33.5	0.5
17.1	0.4	23.9	0.3
11.4	0.3	17.1	0.3
7.6	0.2	11.4	0.1
5.1	0.2	7.6	0.1
		5.1	0.09
$k^S = 17.4 \times 10^{-3}$ $M^{-1}s^{-1}$		$k^S = 17.0 \times 10^{-3}$ $M^{-1}s^{-1}$	
$\Delta k^S_{90\%} = 1.0 \times 10^{-3}$		$\Delta k^S_{90\%} = 2.8 \times 10^{-3}$	
$R^2 = 0.9955$		$R^2 = 0.9648$	

$$k^{\mathrm{MeOH/1000mM\ AcOH}} = (4.8 \pm 3.4)\times 10^{-3}\ M^{-1}s^{-1}$$

$$k^{\mathrm{MeOH/1000mM\ AcOH}} = (17.2 \pm 2.8)\times 10^{-3}\ M^{-1}s^{-1}$$

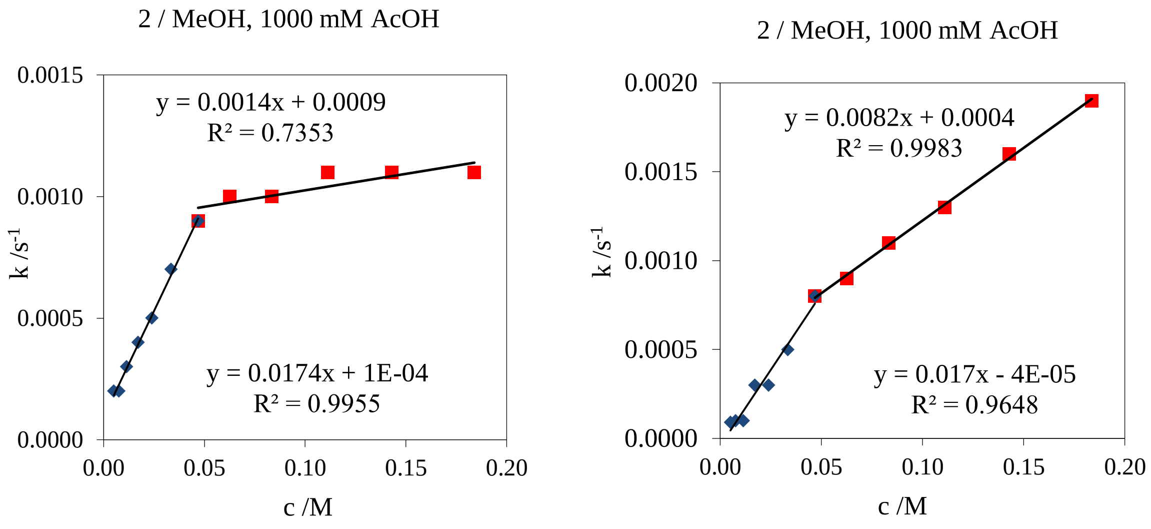

Figure S18. Linear fitting for k_{ex} versus concentration of **2** in methanol /1000 mM AcOH (data from Table S8).

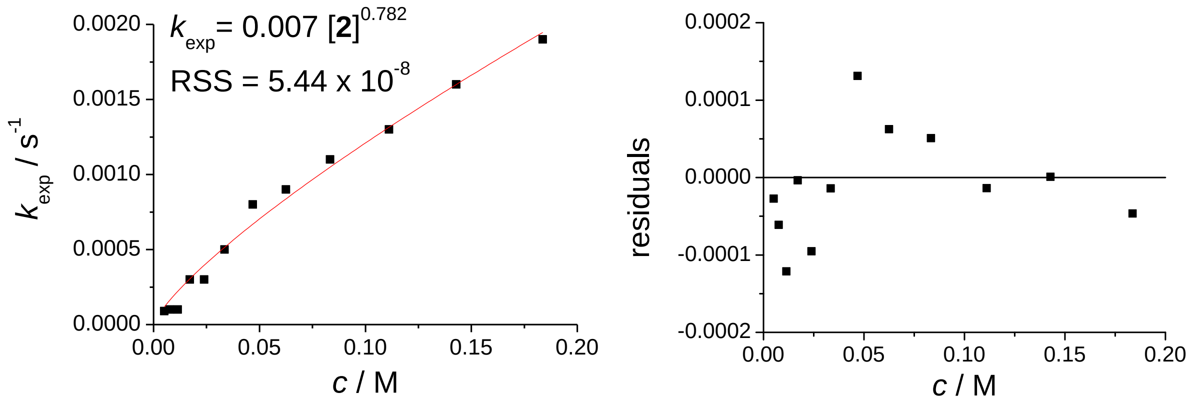

Figure S19. Nonlinear fitting of experimental k_{exp} versus concentration of [**2**] in methanol /1000 mM acetic acid (the set of experimental data from the right column of Table S8) together with residuals of fitting.

Summary of the results for 2 in methanol:

lower concentrations:

k^{MeOH} = (4.4 ± 0.7)×10^{-3} M^{-1}s^{-1}

$k^{MeOH/10mM\ AcOH}$ = (4.0 ± 1.2)×10^{-3} M^{-1}s^{-1}

$k^{MeOH/100mM\ AcOH}$ = (4.5 ± 2.2)×10^{-3} M^{-1}s^{-1}

$k^{MeOH/\ 1000mM\ AcOH}$ = (4.8 ± 3.4)×10^{-3} M^{-1}s^{-1}

higher concentrations

k^{MeOH} = (16.1 ± 2.8)×10^{-3} M^{-1}s^{-1}

$k^{MeOH/10mM\ AcOH}$ = (11.2 ± 0.1)×10^{-3} M^{-1}s^{-1}

$k^{MeOH/100mM\ AcOH}$ = (11.6 ± 2.1)×10^{-3} M^{-1}s^{-1}

$k^{MeOH/\ 1000mM\ AcOH}$ = (17.2 ± 2.8)×10^{-3} M^{-1}s^{-1}

Table S9. Kinetic data for the reaction of **dpph•** with **1** in 1:1 MeOH/water at pH= 5.4. Symbols and abbreviations are the same as in caption to Table S1.

**MeOH/H_2O**, pH = 5.4		**MeOH/H_2O**, pH = 5.4	
[PhOH] /mM	$k_{exp} \times 10^4$ /s^{-1}	[PhOH] /mM	$k_{exp} \times 10^4$ /s^{-1}
138.2	28.7	130.1	26.0
107.5	22.9	101.2	21.7
80.6	17.3	75.9	15.6
60.5	12.7	54.2	11.4
30.8	7.3	38.7	9.7
20.6	6.2	27.6	8.1
13.7	2.9	18.4	6.5
		12.3	3.5
$k^S = 0.20\ M^{-1}s^{-1}$		$k^S = 0.18\ M^{-1}s^{-1}$	
$\Delta k^S_{90\%} = 0.01$		$\Delta k^S_{90\%} = 0.01$	
$R^2 = 0.9959$		$R^2 = 0.99$	
$\boldsymbol{k^{MeOH/pH5.4} = 0.19 \pm 0.03\ M^{-1}s^{-1}}$			

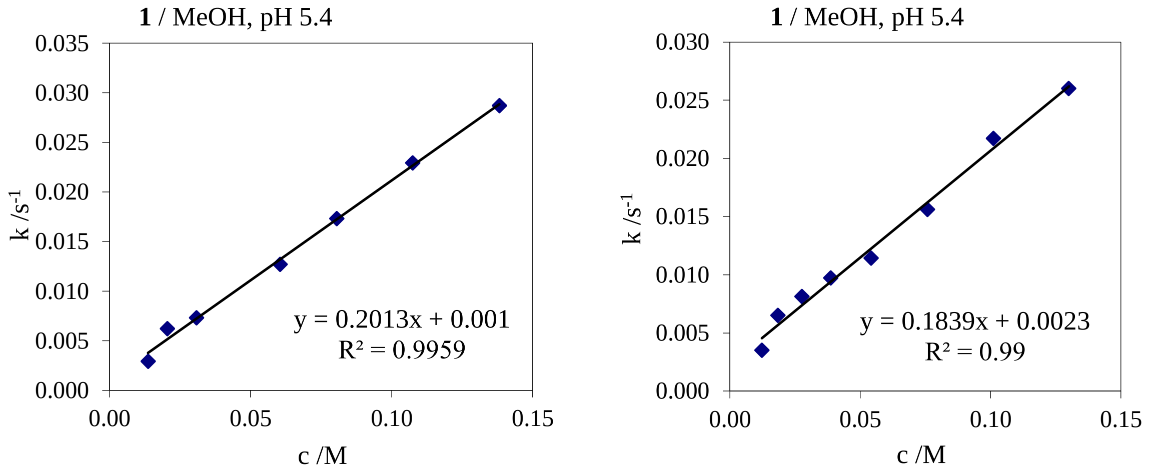

Figure S20. Linear fitting for k_{ex} versus concentration of **1** in methanol/water at pH 5.4 (data from Table S9).

Table S10. Kinetic data for the reaction of **dpph•** with **1** in MeOH/water (1:1) at pH = 7.4. Symbols and abbreviations are the same as in caption to Table S1.

**MeOH/H$_2$O**, pH = 7.4		**MeOH/H$_2$O**, pH = 7.4	
[PhOH] /mM	$k_{ex} \times 10^4$ /s^{-1}	[PhOH] /mM	$k_{ex} \times 10^4$ /s^{-1}
125.1	982	125.0	859
97.3	734	97.2	748
73.0	597	72.9	518
54.7	473	54.7	436
39.1	318	39.1	335
27.9	254	27.9	247
18.6	165	18.6	170
12.4	95	12.4	49
k^S = 0.76 M^{-1}s^{-1}		k^S = 0.71 M^{-1}s^{-1}	
$\Delta k^S_{90\%}$ = 0.04		$\Delta k^S_{90\%}$ = 0.07	
R^2 = 0.9951		R^2 = 0.9786	
$\boldsymbol{k}^{\text{MeOH/pH7.4}}$ **= 0.74 ± 0.07 M^{-1}s^{-1}**			

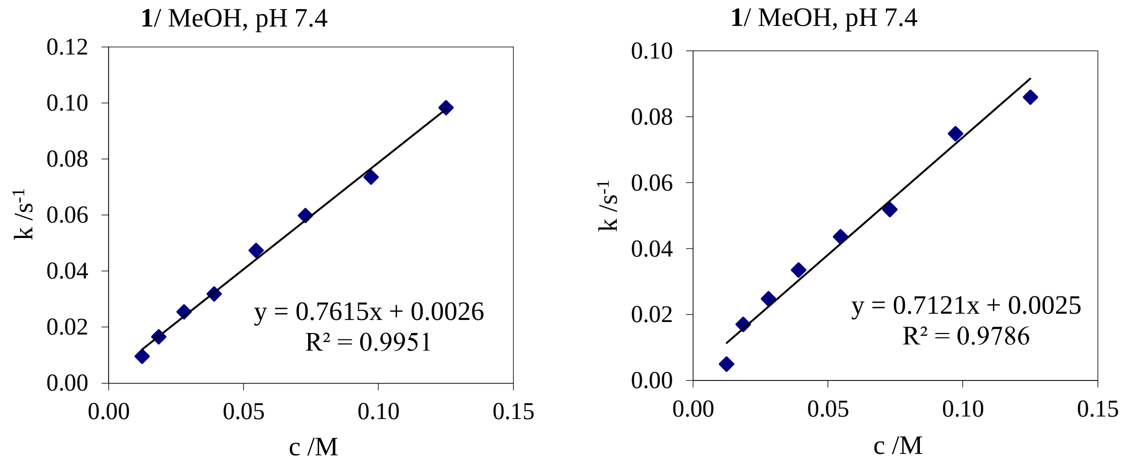

Figure S21. Linear fitting for k_{ex} versus concentration of **1** in methanol /water at pH 7.4 (data from Table S10).

Table S11. Kinetic data for the reaction of **dpph•** with **2** in MeOH/water (1:1) at pH = 5.4. Symbols and abbreviations are the same as in caption to Table S1.

**MeOH/H_2O**, pH = 5.4		**MeOH/H_2O**, pH = 5.4	
[**2**] /mM	$k_{exp} \times 10^4$ /s^{-1}	[**2**] /mM	$k_{exp} \times 10^4$ /s^{-1}
126.9	40.0	126.8	38.1
98.7	30.4	98.7	29.5
74.0	23.3	74.0	24.4
55.5	21.2	55.5	21.6
39.7	17.4	39.6	18.1
28.3	12.8	28.3	13.2
18.9	11.3	18.9	11.5
12.6	8.4	12.6	10.3
k^S = 0.26 $M^{-1}s^{-1}$		k^S = 0.24 $M^{-1}s^{-1}$	
$\Delta k^S_{90\%}$ = 0.03		$\Delta k^S_{90\%}$ = 0.02	
R^2 = 0.9884		R^2 = 0.9906	
$\boldsymbol{k^{MeOH/\ pH\ 5.4}}$ = **0.25± 0.01 $M^{-1}s^{-1}$**			

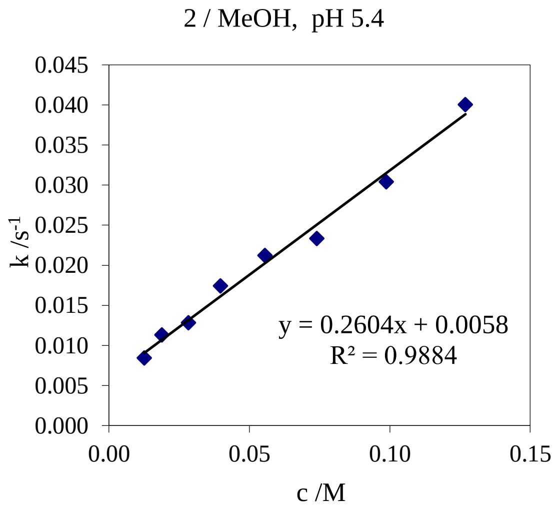

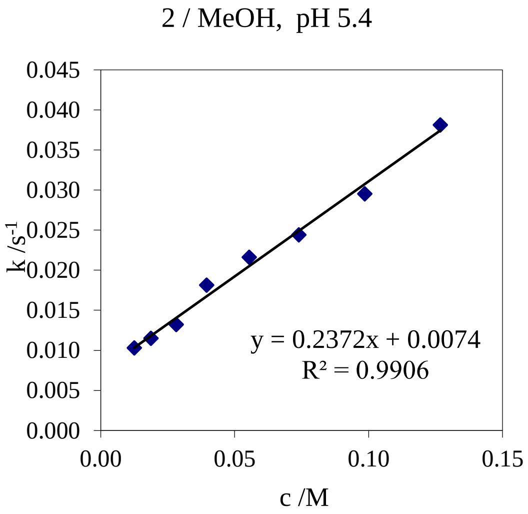

Figure S22. Linear fitting for k_{ex} versus concentration of **2** in methanol /water at pH 5.4 (data from Table S11).

Table S12. Kinetic data for the reaction of **dpph•** with **2** in MeOH/water (1:1) at pH = 7.4. Symbols and abbreviations are the same as in caption to Table S1.

**MeOH/H$_2$O**, pH = 7.4		**MeOH/H$_2$O**, pH = 7.4	
[**2**] /mM	$k_{ex} \times 10^4$ /s^{-1}	[**2**] /mM	$k_{ex} \times 10^4$ /s^{-1}
73.2	786	97.0	858
54.9	751	72.7	736
39.2	636	54.5	723
28.0	541	39.0	600
18.7	437	27.8	506
12.5	364	18.6	436
		12.4	300
$k^S = 0.71$ M^{-1}s^{-1}		$k^S = 0.60$ M^{-1}s^{-1}	
$\Delta k^S_{90\%} = 0.07$		$\Delta k^S_{90\%} = 0.16$	
$R^2 = 0.9395$		$R^2 = 0.9139$	
$\boldsymbol{k^{MeOH/\ pH\ 7.4} = 0.66 \pm 0.12}$ **M^{-1}s^{-1}**			

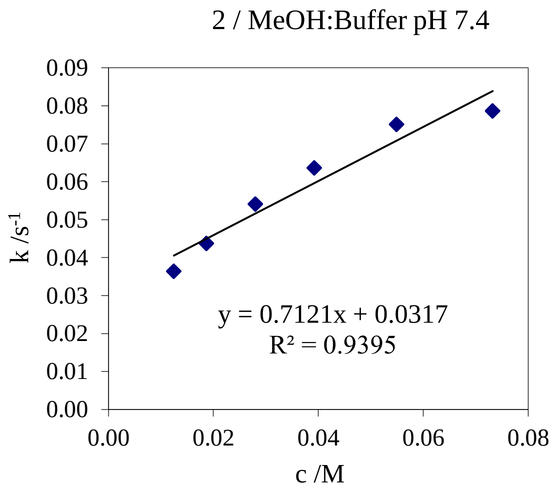

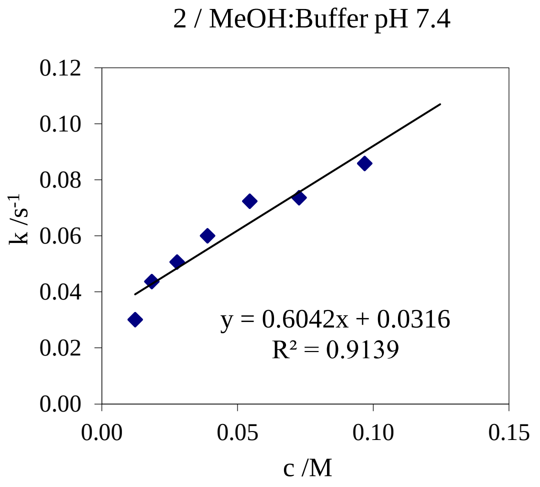

Figure S23. Linear fitting for k_{ex} versus concentration of **2** in methanol/water at pH 7.4 (data from Table S12).

Table S13. Kinetic data for the reaction of **dpph•** with **3** in MeOH. Symbols and abbreviations are the same as in caption to Table S1.

Neat MeOH		Neat MeOH	
[**3**] /mM	$k_{exp} \times 10^3$ /s^{-1}	[**3**] /mM	$k_{exp} \times 10^3$ /s^{-1}
0.80	389.8	0.78	357
0.60	374.4	0.59	323
0.45	360.1	0.44	301
0.34	329	0.33	279
0.25	301.9	0.25	257
0.19	276.6	0.19	231
k^S = 182 M^{-1}s^{-1}		k^S = 198.8 M^{-1}s^{-1}	
$\Delta k^S_{90\%}$ = 60		$\Delta k^S_{90\%}$ = 31.9	
R^2 = 0.8967		R^2 = 0.9734	
0.19	276.6	0.19	231
0.14	229	0.13	188
0.10	192.1	0.10	168
0.06	146.5	0.06	128
0.04	114.6	0.04	89
0.03	70.7	0.03	57
k^S = 1200 M^{-1}s^{-1}		k^S = 1000 M^{-1}s^{-1}	
$\Delta k^S_{90\%}$ = 200		$\Delta k^S_{90\%}$ = 250	
R^2 = 0.9682		R^2 = 0.9488	

$$k^{MeOH} = 190 \pm 10\ M^{-1}s^{-1}$$

$$k^{MeOH} = 1100 \pm 100\ M^{-1}s^{-1}$$

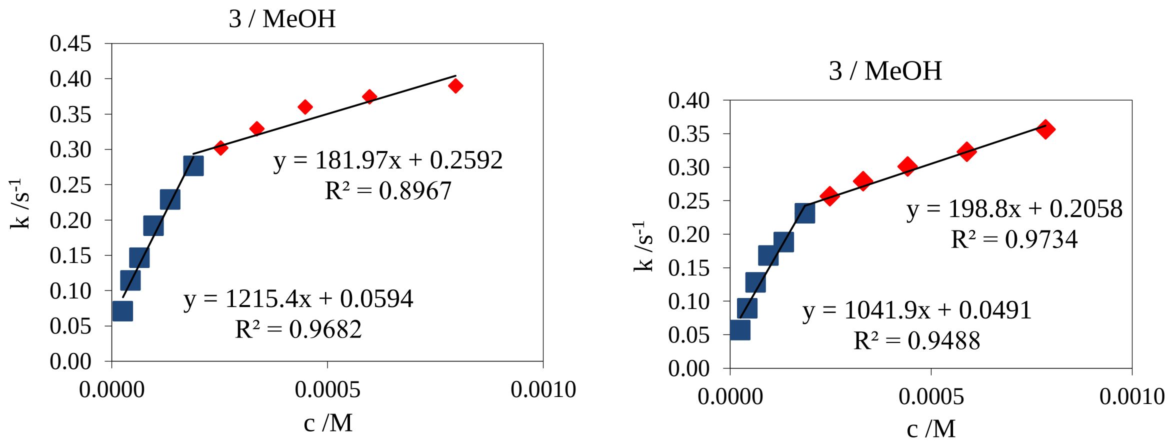

Figure S24. Linear fitting for k_{ex} versus concentration of **3** (data from Table S13).

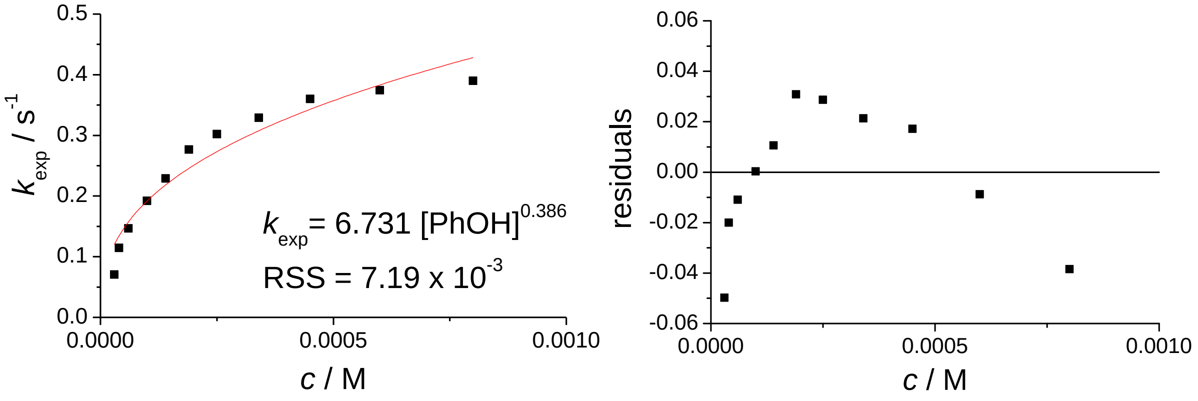

Figure S25. Nonlinear fitting of experimental k_{exp} versus concentration of [**3**] (the set of experimental data from the left column of Table S13) together with residuals of fitting.

Table S14. Kinetic data for the reaction of **dpph•** with **3** in MeOH with AcOH (10 mM). Symbols and abbreviations are the same as in caption to Table S1.

**MeOH/ 10mM AcOH**		**MeOH/ 10mM AcOH**	
[**3**] /mM	$k_{exp} \times 10^3$ /s^{-1}	[**3**] /mM	$k_{exp} \times 10^3$ /s^{-1}
1.03	66.4	1.01	64.2
0.80	55.5	0.79	46.2
0.60	45.4	0.59	35.2
0.32	31.2	0.44	29.1
0.23	23.9	0.33	24.2
0.15	20.5	0.25	15
		0.19	10.8
		0.13	10.5
		0.10	9.3
		0.04	5.2
		0.03	4.9
$k^S = 53$ $M^{-1}s^{-1}$		$k^S = 59$ $M^{-1}s^{-1}$	
$\Delta k^S_{90\%} = 3$		$\Delta k^S_{90\%} = 4$	
$R^2 = 0.9972$		$R^2 = 0.9905$	

$$k^{\text{MeOH/ 10 mM AcOH}} = 56 \pm 4\ M^{-1}s^{-1}$$

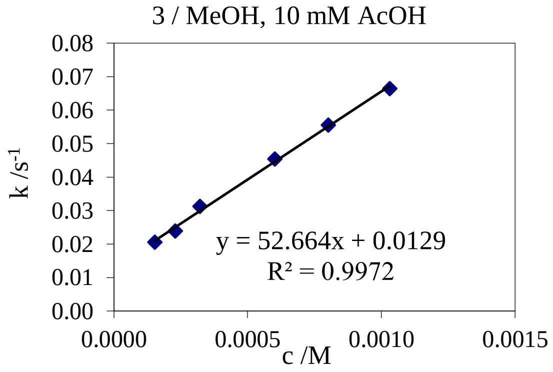

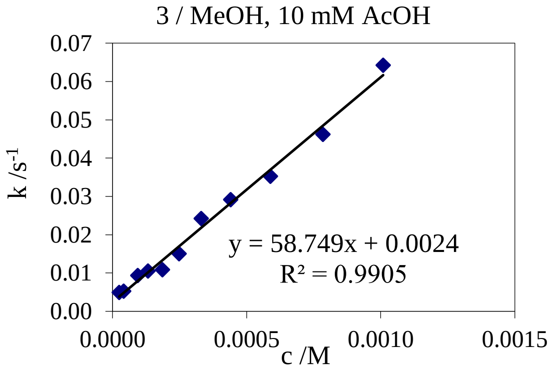

Figure S26. Linear fitting for k_{ex} versus concentration of **3** (data from Table S14).

Table S15. Kinetic data for the reaction of **dpph•** with **3** in MeOH with AcOH (100 mM). Symbols and abbreviations are the same as in caption to Table S1.

**MeOH/ 100mM AcOH**		**MeOH/ 100mM AcOH**	
[**3**] /mM	$k_{exp} \times 10^3$ /s^{-1}	[**3**] /mM	$k_{exp} \times 10^3$ /s^{-1}
1.01	57.1	1.04	56.2
0.79	52	0.81	48.9
0.59	37.1	0.61	38
0.44	33.3	0.45	30.8
0.32	23.6	0.32	21
0.15	15	0.23	18.1
0.10	6.5	0.15	12.6
		0.10	11.1
k^S = 55 M^{-1}s^{-1}		k^S = 51 M^{-1}s^{-1}	
$\Delta k^S_{90\%}$ = 8		$\Delta k^S_{90\%}$ = 4	
R^2 = 0.9702		R^2 = 0.9908	

$$\boldsymbol{k^{\text{MeOH/ 100 mM AcOH}} = 53 \pm 3\ \text{M}^{-1}\text{s}^{-1}}$$

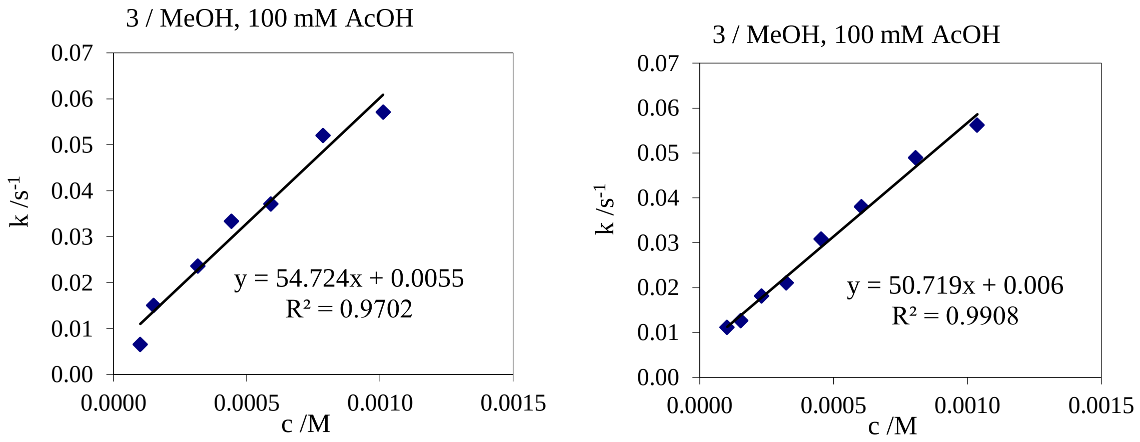

Figure S27. Linear fitting for k_{ex} versus concentration of **3** (data from Table S15).

Table S16. Kinetic data for the reaction of **dpph•** with **3** in MeOH with AcOH (1 M). Symbols and abbreviations are the same as in caption to Table S1.

**MeOH/ 1000mM AcOH**		**MeOH/ 1000mM AcOH**	
[**3**] /mM	$k_{exp} \times 10^3$ /s^{-1}	[**3**] /mM	$k_{exp} \times 10^3$ /s^{-1}
1.04	59.1	1.03	63.1
0.81	43.6	0.80	55.1
0.61	35.0	0.60	38.9
0.45	30.1	0.45	31.8
0.32	19.7	0.32	17.3
0.23	17.3	0.23	13.2
0.15	14.8	0.15	8.8
0.10	12.9		
$k^S = 50$ M^{-1}s^{-1}		$k^S = 66$ M^{-1}s^{-1}	
$\Delta k^S_{90\%} = 4$		$\Delta k^S_{90\%} = 7$	
$R^2 = 0.9882$		$R^2 = 0.9834$	

$$k^{\text{MeOH/ 1000 mM AcOH}} = 57 \pm 12 \text{ M}^{-1}\text{s}^{-1}$$

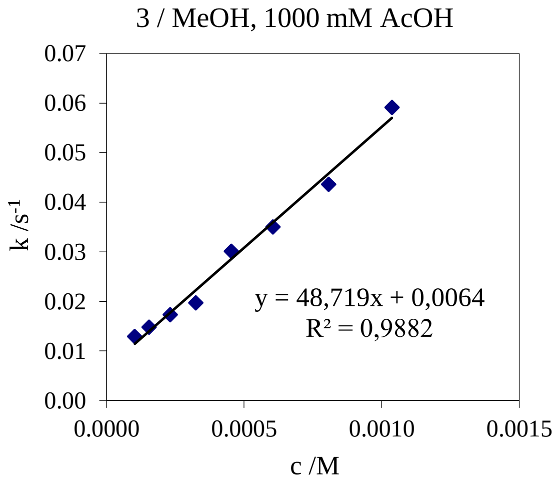

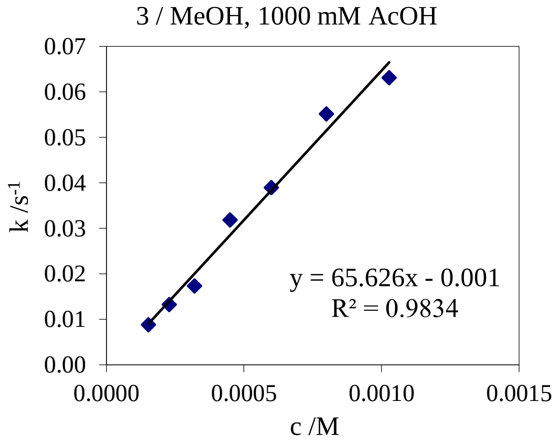

Figure S28. Linear fitting for k_{ex} versus concentration of **3** (data from Table S16).

Summary of the results for **3** in methanol:

$k^{\text{MeOH}} = 190 \pm 10$ M^{-1}s^{-1}

$k^{\text{MeOH}} = 1100 \pm 100$ M^{-1}s^{-1}

$k^{\text{MeOH/ 10 mM AcOH}} = 56 \pm 4$ M^{-1}s^{-1}

$k^{\text{MeOH/ 100 mM AcOH}} = 53 \pm 3$ M^{-1}s^{-1}

$k^{\text{MeOH/ 1000 mM AcOH}} = 57 \pm 12$ M^{-1}s^{-1}

Table S17. Kinetic data for the reaction of **dpph•** with **4** in MeOH. Symbols and abbreviations are the same as in caption to Table S1.

**Neat MeOH**		**Neat MeOH**	
[**4**] /mM	$k_{exp} \times 10^3$ /s^{-1}	[**4**] /mM	$k_{exp} \times 10^3$ /s^{-1}
0.72	579.3	0.72	629.8
0.54	548.9	0.54	574.9
0.40	519.1	0.40	510.6
0.30	472.9	0.30	463.3
0.23	427.0		
k^S = 300 M^{-1}s^{-1}		k^S = 400 M^{-1}s^{-1}	
$\Delta k^S_{90\%}$ = 100		$\Delta k^S_{90\%}$ = 70	
R^2 = 0.9273		R^2 = 0.9877	
0.23	427.0	0.30	463.3
0.17	384.8	0.23	391.8
0.12	330.3	0.17	341.6
0.09	280.0	0.12	292.9
0.06	216.9	0.09	232.9
0.04	165.6	0.06	180.7
0.02	121.4	0.04	127.4
		0.02	96.1
k^S = 1500 M^{-1}s^{-1}		k^S = 1300 M^{-1}s^{-1}	
$\Delta k^S_{90\%}$ = 300		$\Delta k^S_{90\%}$ = 200	
R^2 = 0.9452		R^2 = 0.9606	

$$k^{MeOH} = 350 \pm 50\ M^{-1}s^{-1}$$
$$k^{MeOH} = 1400 \pm 100\ M^{-1}s^{-1}$$

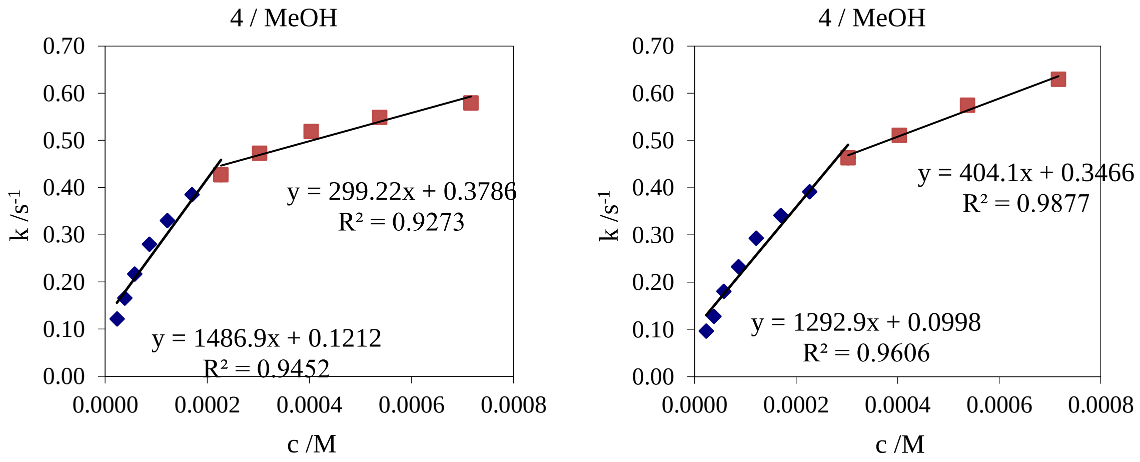

Figure S29. Linear fitting for k_{ex} versus concentration of **4** (data from Table S17).

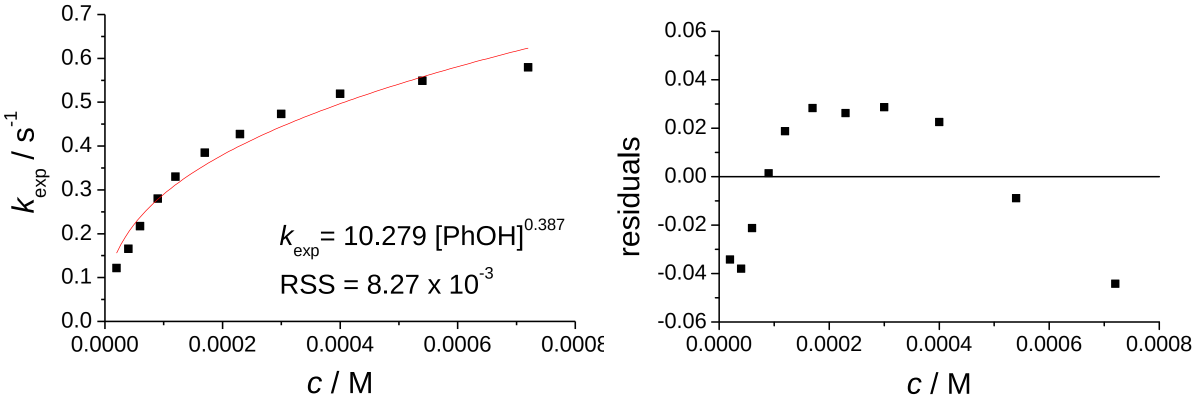

Figure S30. Nonlinear fitting of experimental k_{exp} versus concentration of [**4**] (the set of experimental data from the left column of Table S17) together with residuals of fitting.

Table S18. Kinetic data for the reaction of **dpph•** with **4** in MeOH with AcOH (10 mM). Symbols and abbreviations are the same as in caption to Table S1.

**MeOH/ 10mM AcOH**		**MeOH/ 10mM AcOH**	
[**4**] /mM	$k_{exp} \times 10^3$ /s^{-1}	[**4**] /mM	$k_{exp} \times 10^3$ /s^{-1}
0.91	75.20	0.70	65.00
0.71	62.10	0.52	49.10
0.53	55.70	0.29	32.80
0.40	46.10	0.22	27.20
0.30	38.60	0.17	20.90
0.23	34.10	0.12	15.40
0.17	25.10	0.08	15.50
0.12	18.20	0.06	14.50
0.06	12.70	0.02	6.20
0.04	13.90		
k^S = 72 M^{-1}s^{-1}		k^S = 83 M^{-1}s^{-1}	
$\Delta k^S_{90\%}$ = 9		$\Delta k^S_{90\%}$ = 5	
R^2 = 0.9687		R^2 = 0.9914	

$$\boldsymbol{k^{\text{MeOH/ 10mM AcOH}} = 77 \pm 5\ M^{-1}s^{-1}}$$

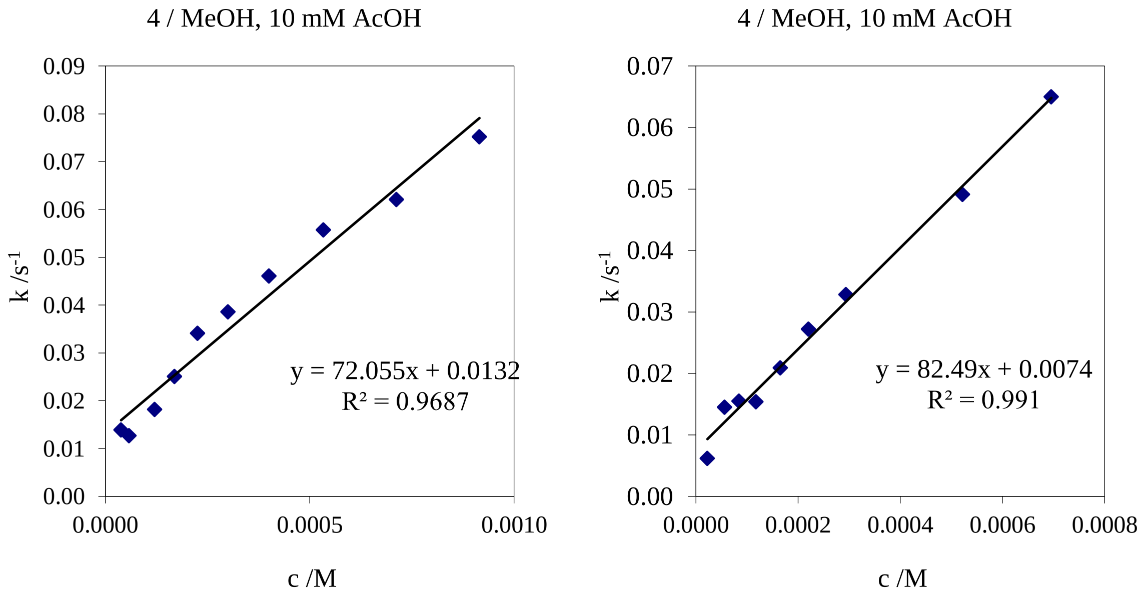

Figure S31. Linear fitting for k_{ex} versus concentration of **4** (data from Table S18).

Table S19. Kinetic data for the reaction of **dpph•** with **4** in MeOH with AcOH (100 mM). Symbols and abbreviations are the same as in caption to Table S1.

**MeOH/ 100mM AcOH**		**MeOH/ 100mM AcOH**	
[**4**] /mM	$k_{exp} \times 10^3$ /s^{-1}	[**4**] /mM	$k_{exp} \times 10^3$ /s^{-1}
0.71	52.8	0.92	60.5
0.54	33.8	0.71	48.4
0.40	31.5	0.53	38.7
0.29	18.9	0.40	26.2
0.21	11.9	0.29	21.2
0.14	10.2	0.20	19.3
0.09	8.1	0.14	14.5
		0.09	9.6
k^S = 72 M^{-1}s^{-1}		k^S = 61 M^{-1}s^{-1}	
$\Delta k^S_{90\%}$ = 10		$\Delta k^S_{90\%}$ = 4	
R^2 = 0.9730		R^2 = 0.9916	
$\boldsymbol{k}$ **MeOH/ 100mM AcOH = 66 ± 6 M^{-1}s^{-1}**			

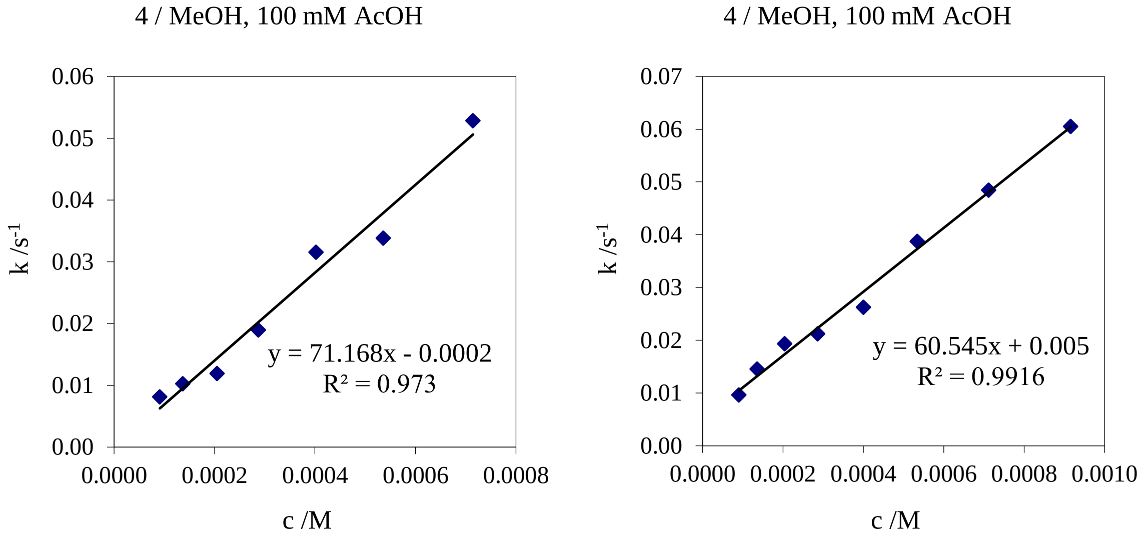

Figure S32. Linear fitting for k_{ex} versus concentration of **4** (data from Table S19).

Table S20. Kinetic data for the reaction of **dpph•** with **4** in MeOH with AcOH (1 M). Symbols and abbreviations are the same as in caption to Table S1.

**MeOH/ 1000mM AcOH**		**MeOH/ 1000mM AcOH**	
[**4**] /mM	$k_{exp} \times 10^3$ /s^{-1}	[**4**] /mM	$k_{exp} \times 10^3$ /s^{-1}
0.90	51.5	0.71	36.6
0.70	43.0	0.53	28.2
0.53	40.0	0.40	20.8
0.40	34.2	0.28	17.3
0.28	19.7	0.20	15.3
0.20	19.8	0.14	11.6
0.13	13.1	0.09	10.2
k^S = 50 M^{-1}s^{-1}		k^S = 42 M^{-1}s^{-1}	
$\Delta k^S_{90\%}$ = 11		$\Delta k^S_{90\%}$ = 4	
R^2 = 0.936		R^2 = 0.9897	

$k^{\text{MeOH/ 1000mM AcOH}}$ **= 46 ± 5 M^{-1}s^{-1}**

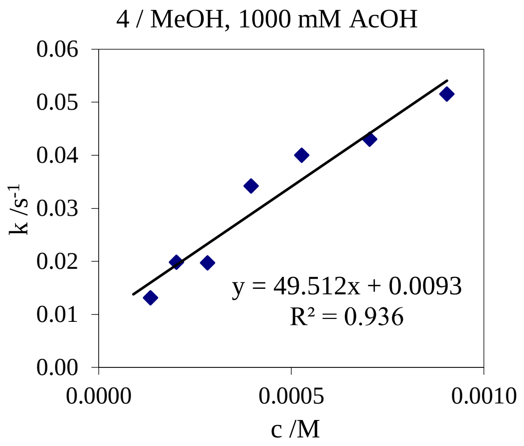

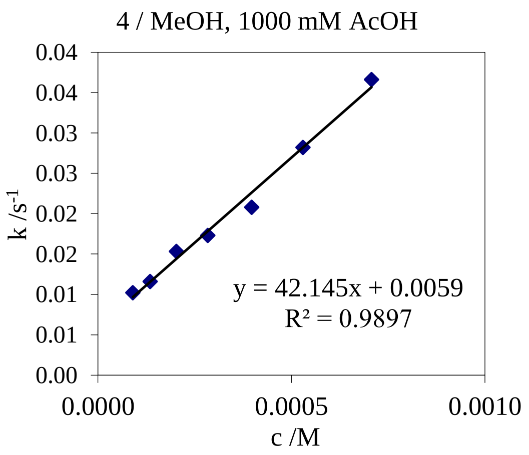

Figure S33. Linear fitting for k_{ex} versus concentration of **4** (data from Table S20).

Summary of the results for **4** in methanol

k^{MeOH} = 350 ± 50 M^{-1}s^{-1}

k^{MeOH} = 1400 ± 100 M^{-1}s^{-1}

$k^{\text{MeOH/ 10mM AcOH}}$ = 77 ± 5 M^{-1}s^{-1}

$k^{\text{MeOH/ 100mM AcOH}}$ = 66 ± 6 M^{-1}s^{-1}

$k^{\text{MeOH/ 1000mM AcOH}}$ = 46 ± 5 M^{-1}s^{-1}

Table S21. Kinetic data for the reaction of **dpph•** with **5** in MeOH. Symbols and abbreviations are the same as in caption to Table S1.

Neat MeOH		Neat MeOH	
[**5**] /mM	$k_{exp} \times 10^3$ /s^{-1}	[**5**] /mM	$k_{exp} \times 10^3$ /s^{-1}
1.27	1906	1.28	1884
0.99	1601	1.00	1686
0.77	1468	0.78	1345
0.58	1134	0.58	1178
0.43	1067	0.44	1016
k^S = 1030 M^{-1}s^{-1}		k^S = 1060 M^{-1}s^{-1}	
$\Delta k^S_{90\%}$ = 180		$\Delta k^S_{90\%}$ = 150	
R^2 = 0.9794		R^2 = 0.9855	
0.43	1067	0.44	1016
0.32	942	0.33	817
0.23	708	0.23	675
0.17	583	0.17	529
0.11	420	0.11	451
0.07	367	0.07	387
k^S = 2050 M^{-1}s^{-1}		k^S = 1750 M^{-1}s^{-1}	
$\Delta k^S_{90\%}$ = 250		$\Delta k^S_{90\%}$ = 80	
R^2 = 0.9844		R^2 = 0.9982	

$$k^{MeOH} = 1050 \pm 15\ M^{-1}s^{-1}$$
$$k^{MeOH} = 1900 \pm 200\ M^{-1}s^{-1}$$

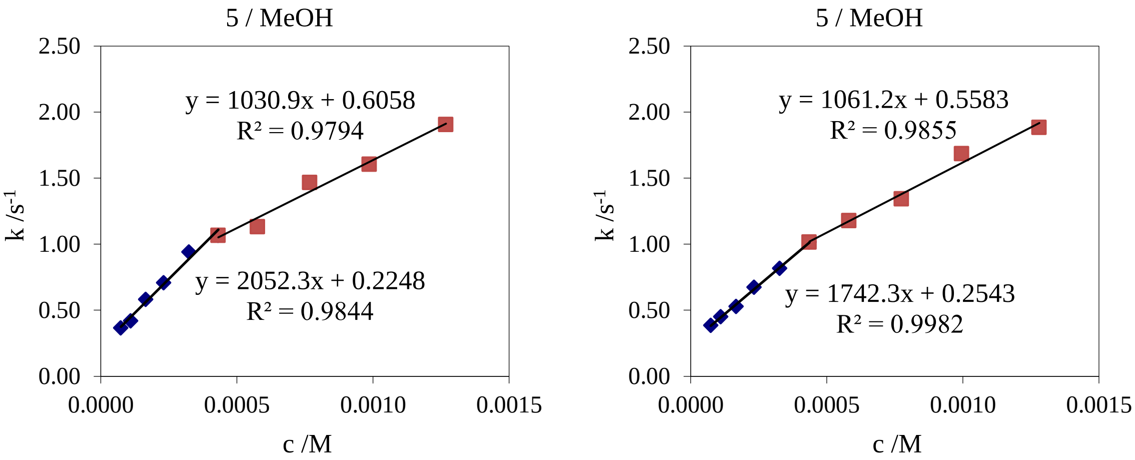

Figure S34. Linear fitting for k_{ex} versus concentration of **5** (data from Table S21).

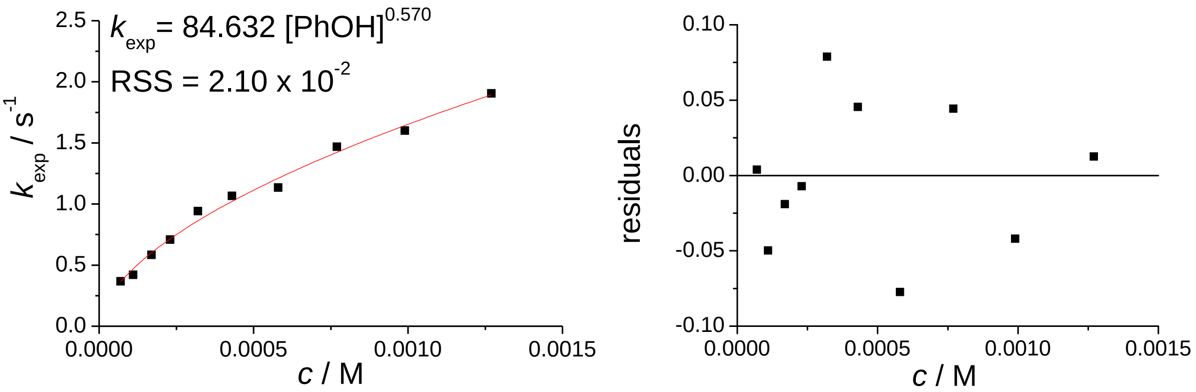

Figure S35. Nonlinear fitting of experimental k_{exp} versus concentration of [5**]** (the set of experimental data from the left column of Table S21) together with residuals of fitting.

Table S22. Kinetic data for the reaction of **dpph•** with **5** in MeOH with AcOH (10 mM). Symbols and abbreviations are the same as in caption to Table S1.

**MeOH/ 10mM AcOH**		**MeOH/ 10mM AcOH**	
[**5**] /mM	$k_{exp} \times 10^3$ /s^{-1}	[**5**] /mM	$k_{exp} \times 10^3$ /s^{-1}
1.29	317.0	1.28	261.2
1.00	268.3	0.99	205.0
0.78	212.3	0.78	163.0
0.59	174.2	0.58	130.0
0.44	148.0	0.44	111.3
0.33	105.0	0.33	99.5
0.23	77.4	0.23	77.0
0.17	60.0	0.17	63.5
0.12	41.9	0.11	42.6
k^S = 240 M^{-1}s^{-1}<br>$\Delta k^S_{90\%}$ = 20<br>R^2 = 0.9886		k^S = 180 M^{-1}s^{-1}<br>$\Delta k^S_{90\%}$ = 10<br>R^2 = 0.9934	

$$\boldsymbol{k}^{\text{MeOH/ 10mM AcOH}} = \mathbf{210 \pm 40\ M^{-1}s^{-1}}$$

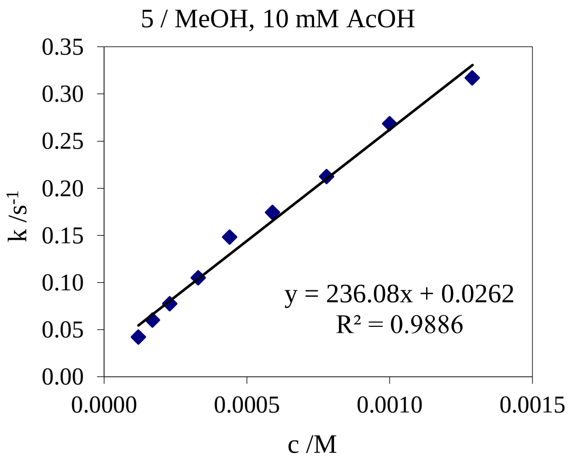

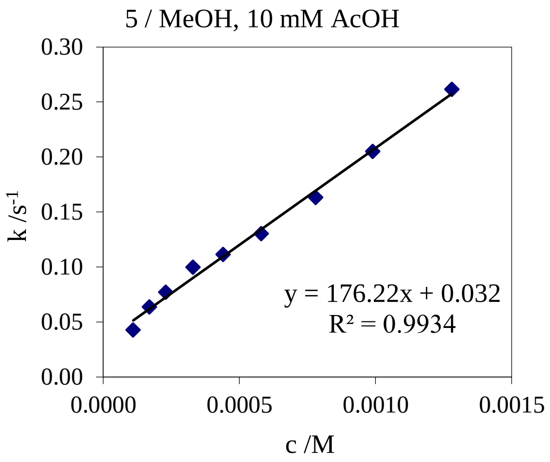

Figure S36. Linear fitting for k_{ex} versus concentration of **5** (data from Table S22).

Table S23. Kinetic data for the reaction of **dpph•** with **5** in MeOH with AcOH (100 mM). Symbols and abbreviations are the same as in caption to Table S1.

**MeOH/ 100mM AcOH**		**MeOH/ 100mM AcOH**	
[**5**] /mM	$k_{exp} \times 10^3$ /s^{-1}	[**5**] /mM	$k_{exp} \times 10^3$ /s^{-1}
1.26	59.5	1.26	54.4
0.98	51.9	0.98	47.8
0.76	39.3	0.57	33.6
0.57	33.5	0.43	27.3
0.43	30.5	0.32	24.9
0.32	23.6	0.23	19.3
0.16	19.2	0.17	14.9
0.11	18.0	0.11	12.2
0.07	16.3	0.07	10.9
k^S = 37 M^{-1}s^{-1}		k^S = 37 M^{-1}s^{-1}	
$\Delta k^S_{90\%}$ = 3		$\Delta k^S_{90\%}$ = 4	
R^2 = 0.9899		R^2 = 0.9831	

$$\boldsymbol{k}^{\text{MeOH/ 100mM AcOH}} = \mathbf{37 \pm 1\ M^{-1}s^{-1}}$$

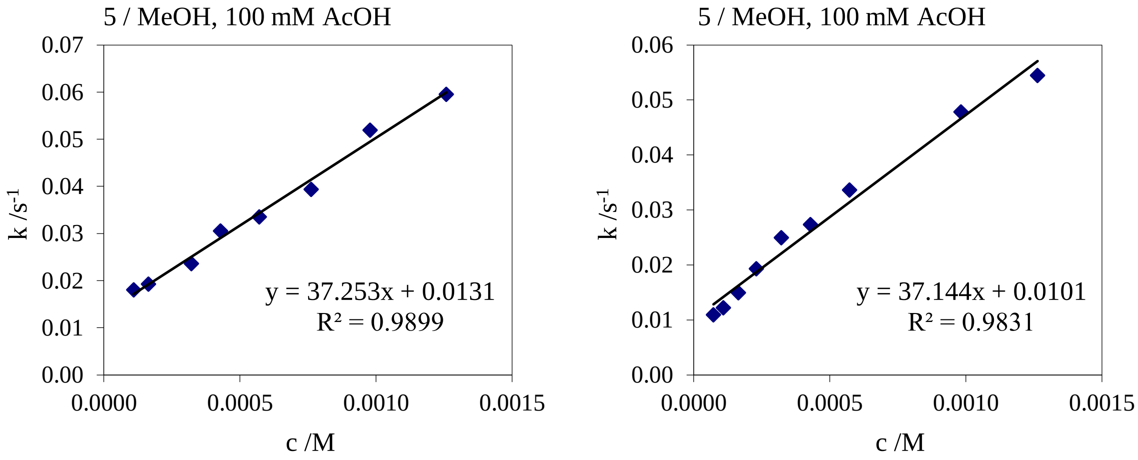

Figure S37. Linear fitting for k_{ex} versus concentration of **5** (data from Table S23).

Table S24. Kinetic data for the reaction of **dpph•** with **5** in MeOH with AcOH (1 M). Symbols and abbreviations are the same as in caption to Table S1.

**MeOH/ 1000mM AcOH**		**MeOH/ 1000mM AcOH**	
[**5**] /mM	$k_{exp} \times 10^3$ /s^{-1}	[**5**] /mM	$k_{exp} \times 10^3$ /s^{-1}
1.24	39.5	1.26	47.5
0.96	32.5	0.98	43.9
0.75	29.9	0.76	37.4
0.56	25.5	0.57	31.0
0.42	23.4	0.43	27.5
0.32	21.5	0.23	24.0
0.23	19.3	0.17	22.0
0.16	17.4	0.11	19.8
0.11	14.7		
k^S = 21 $M^{-1}s^{-1}$		k^S = 25 $M^{-1}s^{-1}$	
$\Delta k^S_{90\%}$ = 2		$\Delta k^S_{90\%}$ = 2	
R^2 = 0.9875		R^2 = 0.9885	

$$k^{\text{MeOH/ 1000mM AcOH}} = 23 \pm 1\ M^{-1}s^{-1}$$

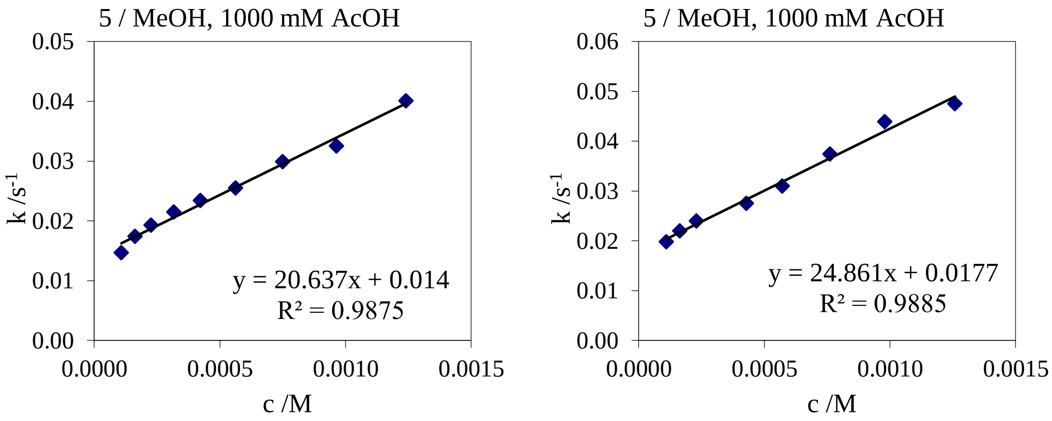

Figure S38. Linear fitting for k_{ex} versus concentration of **5** (data from Table S24).

Summary of the results for **5** in methanol

k^{MeOH} = 1050 ± 15 $M^{-1}s^{-1}$

k^{MeOH} = 1900 ± 200 $M^{-1}s^{-1}$

$k^{\text{MeOH/ 10mM AcOH}}$ = 210 ± 40 $M^{-1}s^{-1}$

$k^{\text{MeOH/ 100mM AcOH}}$ = 37 ± 1 $M^{-1}s^{-1}$

$k^{\text{MeOH/ 1000mM AcOH}}$ = 23 ± 3 $M^{-1}s^{-1}$

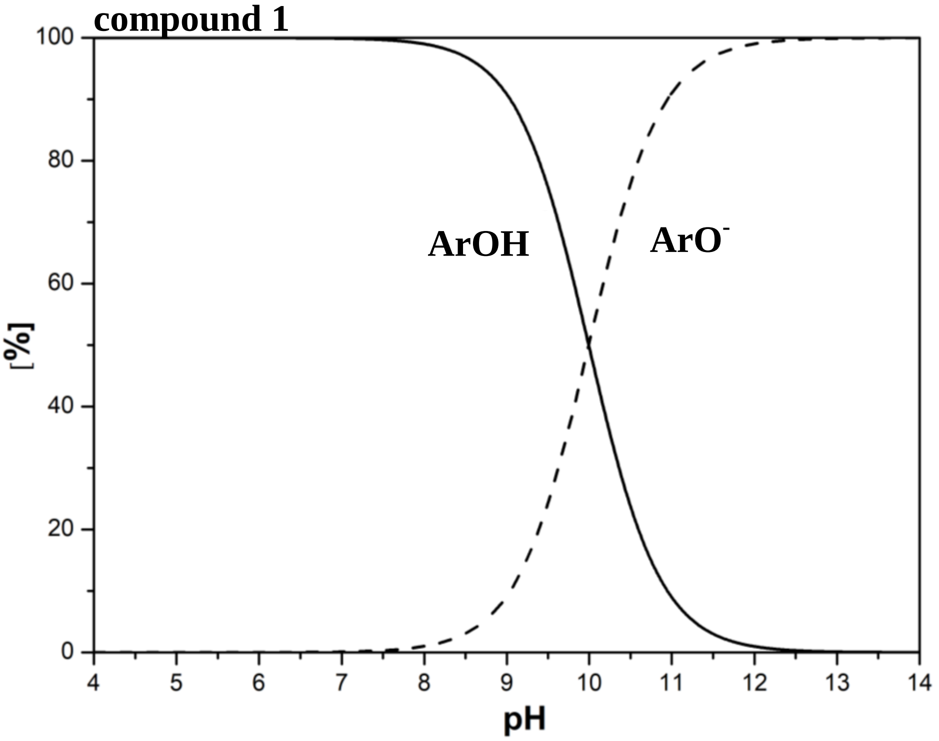

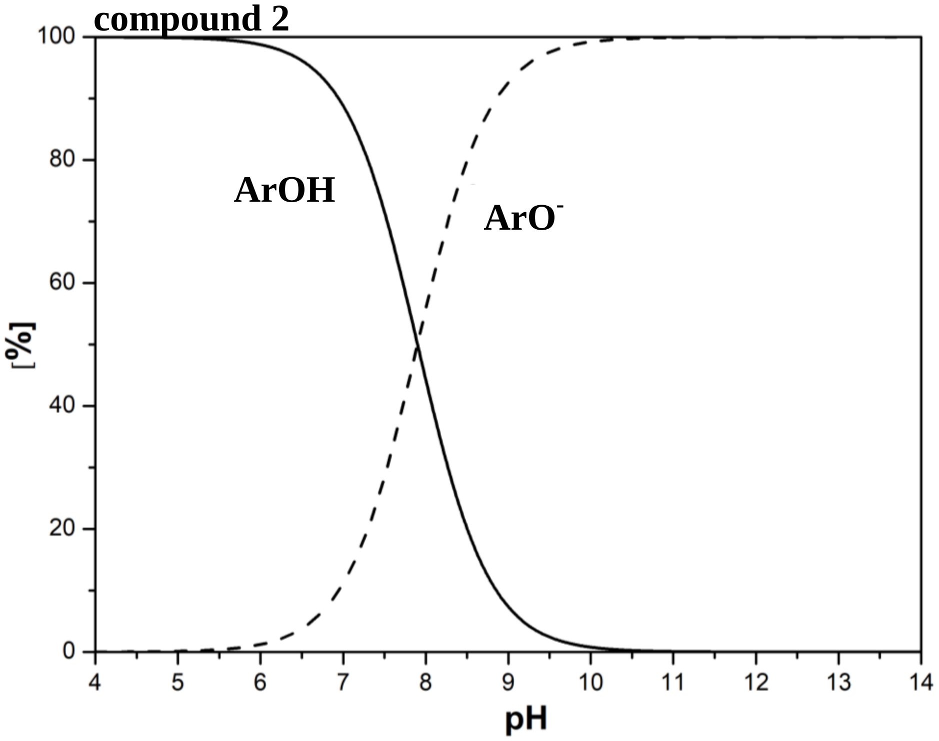

Figure S39. Dissociation diagrams of **1** (upper panel) and **2** (lower panel) within the pH range 4−14 (in water) calculated with pK_a values listed in Table 1 of the main manuscript. Solid line – neutral form, ArOH; dashed line – monoanion, ArO^-.

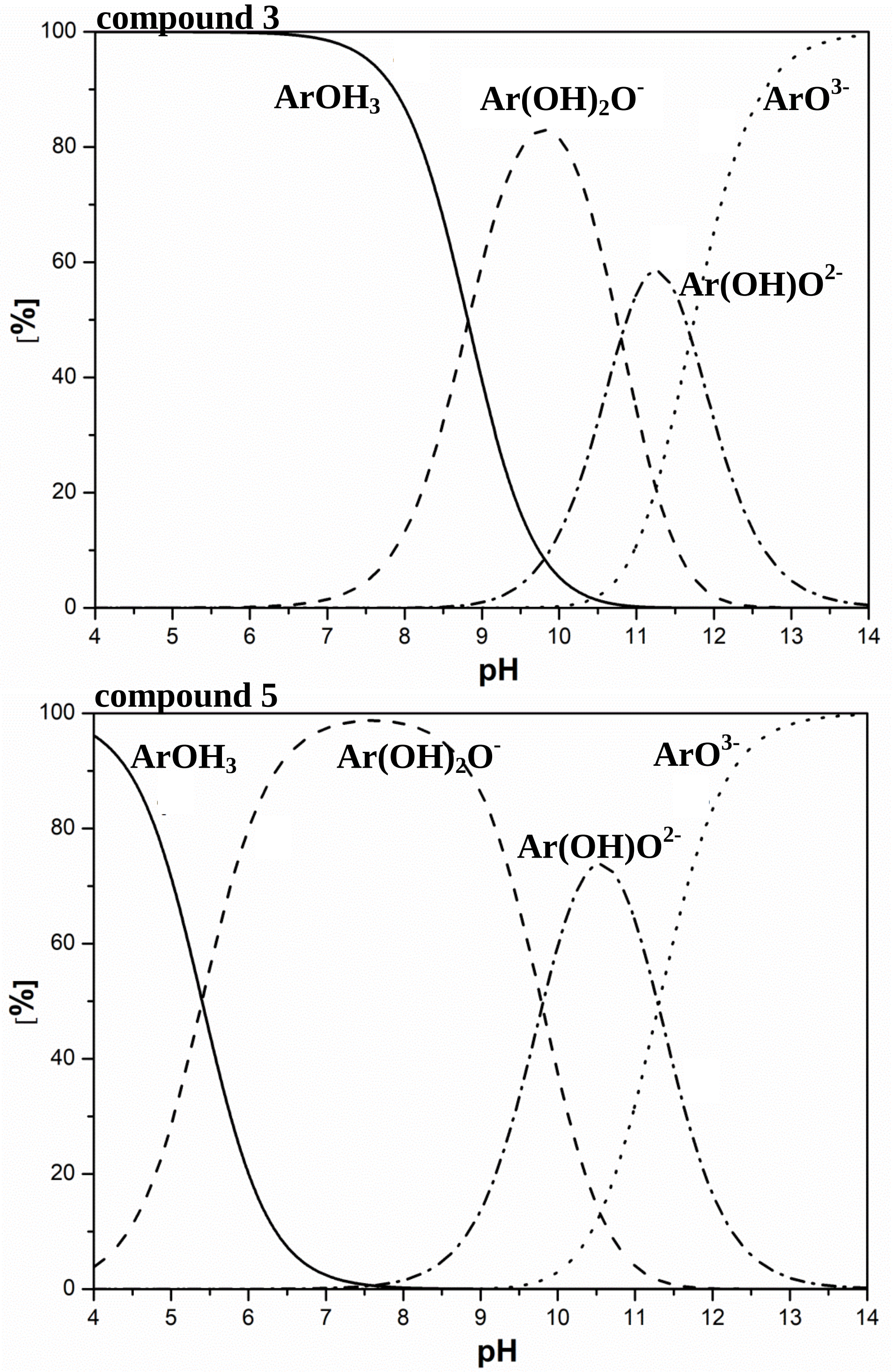

Figure S40. Dissociation diagrams of **3** (upper panel) and **5** (lower panel) within the pH range 4−14 (in water) calculated with pK_a values listed in Table 1 of the main manuscript. Solid line – neutral form, $Ar(OH)_3$; dashed line – monoanion, $Ar(OH)_2O^-$; dotted-dashed line – dianion, $Ar(OH)O^{2-}$; dotted line - trianion, ArO^{3-} , respectively (three-step dissociation was calculated for both compounds).

Estimation of the rate constants for electron transfer from phenolate anions to dpph$^{\bullet}$.

The rate law for the reaction:

$$ArOH + dpph^{\bullet} \rightarrow ArO^{\bullet} + dpph\text{-}H \quad ; \text{overall } k^{S} \qquad (S1)$$

carried out in pseudo first-order conditions (excess of [ArOH] over [dpph$^{\bullet}$]) is:

$$-d[dpph^{\bullet}]/dt = n\, k^{S}\, [ArOH]\, [dpph^{\bullet}] = n\, k_{exp}\, [dpph^{\bullet}] \qquad (S2)$$

The rates for two different concentrations $[ArOH]_1$ and $[ArOH]_1$ can be compared:

$$\frac{R_1}{R_2} = \frac{(-d[dpph^{\bullet}]/dt)_1}{(-d[dpph^{\bullet}]/dt)_2} = \frac{n k_{exp1}\,[dpph^{\bullet}]}{n k_{exp2}\,[dpph^{\bullet}]} \approx \frac{k_{exp1}}{k_{exp2}} \qquad (S3)$$

The rate low for the overall process is a sum of two processes, HAT and ET:

$$R = -\frac{d[dpph^{\bullet}]}{dt} = n(k^{HAT}\,[ArOH] + k^{ET}\,[ArO^{-}])\,[dpph^{\bullet}] \qquad (S4)$$

combination of S3 and S4 gives:

$$\frac{k_{exp1}}{k_{exp2}} \approx \frac{n(k^{HAT}\,[ArOH]_1 + k^{ET}\,[ArO^{-}]_1)\,[dpph^{\bullet}]}{n(k^{HAT}\,[ArOH]_2 + k^{ET}\,[ArO^{-}]_2)\,[dpph^{\bullet}]} = \frac{k^{HAT}\,[ArOH]_1 + k^{ET}\,[ArO^{-}]_1}{k^{HAT}\,[ArOH]_2 + k^{ET}\,[ArO^{-}]_2} \qquad (S5)$$

For very small degree of dissociation: $[ArOH]-[ArO^{-}] \approx [ArOH]$, and substituting

$[ArO^{-}]_1 = \sqrt{K_a[ArOH]_1}$ and $[ArO^{-}]_2 = \sqrt{K_a[ArOH]_2}$:

$$\frac{k_{exp1}}{k_{exp2}} = \frac{k^{HAT}\,[ArOH]_1 + k^{ET}\sqrt{K_a[ArOH]_1}}{k^{HAT}\,[ArOH]_2 + k^{ET}\sqrt{K_a[ArOH]_2}} \qquad (S6)$$

$$k_{exp1}k^{HAT}\,[ArOH]_2 + k_{exp1}k^{ET}\sqrt{K_a[ArOH]_2} = k_{exp2}k^{HAT}\,[ArOH]_1 + k_{exp2}k^{ET}\sqrt{K_a[ArOH]_1} \qquad (S7)$$

$$k_{exp1}k^{HAT}\,[ArOH]_2 - k_{exp2}k^{HAT}\,[ArOH]_1 = k_{exp2}k^{ET}\sqrt{K_a[ArOH]_1} - k_{exp1}k^{ET}\sqrt{K_a[ArOH]_2} \qquad (S8)$$

$$k^{HAT}\left(k_{exp1}[ArOH]_2 - k_{exp2}\,[ArOH]_1\right) = k^{ET}\sqrt{K_a}\left(k_{exp2}\sqrt{[ArOH]_1} - k_{exp1}\sqrt{[ArOH]_2}\right) \qquad (S9)$$

$$\frac{k^{ET}}{k^{HAT}} = \frac{(k_{exp1}[ArOH]_2 - k_{exp2}\,[ArOH]_1)}{\sqrt{K_a}\left(k_{exp2}\sqrt{[ArOH]_1} - k_{exp1}\sqrt{[ArOH]_2}\right)} \qquad (S10)$$

Comments:

a) the above proportion is approximate, non-analytical comparison of two processes which exhibit different orders in [ArOH],

b) for very diluted solution of ArOH the errors of measurements of k_{exp} (regardless their origin, like accuracy of signal detection or presence of traces of acids/bases in a solvent) might results in large errors in final k^{ET}/k^{HAT}.

Table S25. Parameters used for estimation of k^{ET}/k^{HAT} ratio.

phenol	1	2	3	5	quercetin[a]
pK_a (MeOH)	14.4	12.2	13.5	9.5	12.8
$[ArOH]_1$ /M	0.150	0.0486	1.9×10^{-4}	4.4×10^{-4}	5.8×10^{-4}
k_{exp1} /s^{-1}	0.0113	0.0009	0.277	1.0116	2.31
$[ArOH]_2$ /M	0.015	0.0014	0.3×10^{-4}	0.7×10^{-4}	0.51×10^{-4}
k_{exp1} /s^{-1}	0.0016	0.00007	0.071	0.387	0.67
k^{ET}/k^{HAT}	1.46×10^{6}	1.48×10^{5}	5.41×10^{4}	1.62×10^{4}	1.68×10^{6}

[a] pK_a (H2O)=8.45 was recalculated to pK_a(MeOH) using correlation described in main manuscript Table 1 (footnote *e*) and k_{exp} for quercetin are taken from our previous publication on flavonoids.[5a]

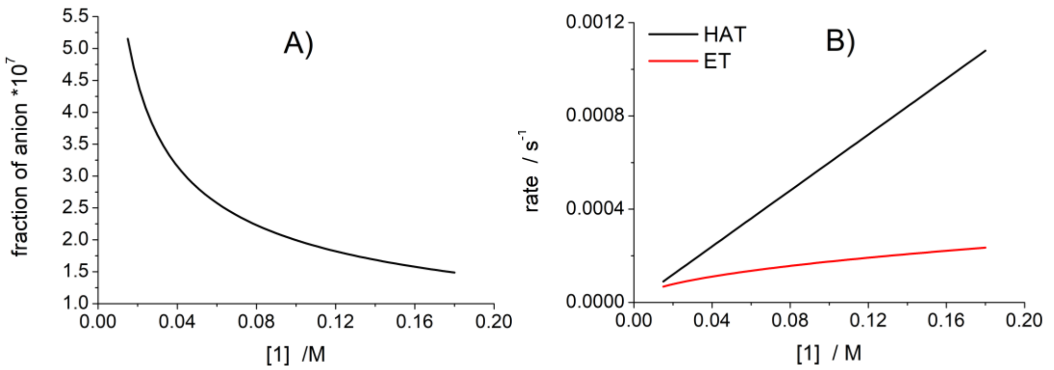

Figure S41. A) Fraction [**1**$^-$]/[**1**] plotted versus [**1**] within the concentration range 15 - 160 mM used in the kinetic experiments. The order of magnitude of fraction is -7. B) Contribution of HAT and ET to the overall rate of reaction of **1** (and **1**$^-$) with dpph$^\bullet$ in methanol. *HAT process dominates within the whole concentration range tested.*

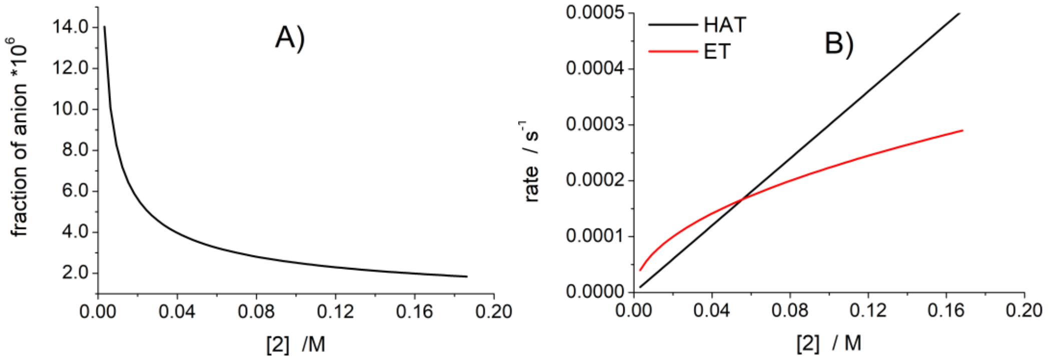

Figure S42. A) Fraction [**2**$^-$]/[**2**] plotted versus [**2**] within the concentration range 32 - 180 mM used in the kinetic experiments. The order of magnitude of fraction is -6. B) Contribution of HAT and ET to the overall rate of reaction of **2**(and **2**$^-$) with dpph$^\bullet$ in methanol. *The intersection is between 50 and 60 mM, ET dominates below this concentration of **2**, while HAT dominates at [**2**] >60 mM.*

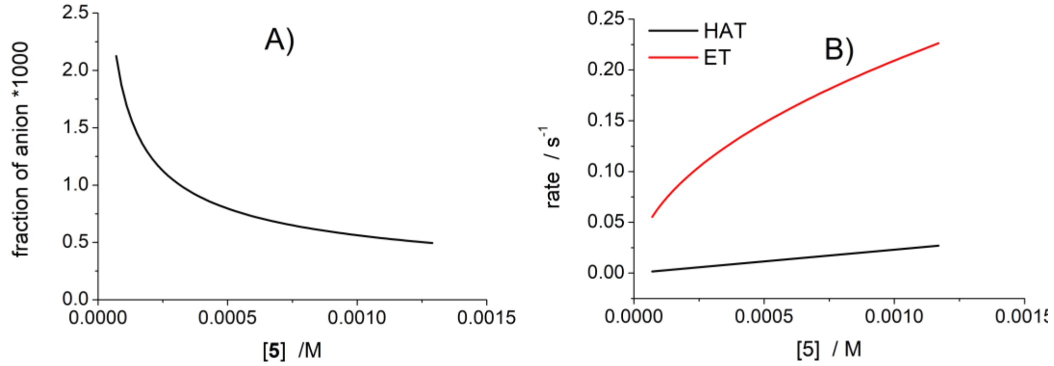

Figure S43. A) Fraction [**5**$^-$]/[**5**] plotted versus [**5**] within the concentration range 0.07 - 1.3 mM used in the kinetic experiments. The order of magnitude of fraction is -3. B) Contribution of HAT and ET to the overall rate of reaction of **5** (and **5**$^-$) with dpph$^\bullet$ in methanol. *Within the whole concentration range ET is a dominating mechanism.*

REFERENCES

1. F. Weinelt and H. J. Schneider, *J. Org. Chem.*, 1991, **56**, 5527-5535.
2. P. Ziaja, A. Krogul, T. S. Pawłowski and G. Litwinienko, *Thermochim. Acta*, 2016, **623**, 112-119.
3. a) D. J. Cram, S. Karbach, H. E. Kim, C. B. Knobler, E. F. Maverick, J. L. Ericson and R. C. Helgeson, *J. Am. Chem. Soc.*, 1988, **110**, 2229-2237; b) Y. Aoyama, Y. Tanaka and S. Sugahara, *J. Am. Chem. Soc.*, 1989, **111**, 5397-5404; c) A. S. Hoegberg, *J. Am. Chem. Soc.*, 1980, **102**, 6046-6050.
4. R. Kuzmicz, V. Kowalska, S. Domagała, M. Stachowicz, K. Woźniak and W. Kolodziejski, *J. Phys. Chem. B*, 2010, **114**, 10311-10320.
5. a) M. Musialik, R. Kuzmicz, T. S. Pawlowski and G. Litwinienko, *J. Org. Chem.*, 2009, **74**, 2699-2709; b) E. Van Wenum, R. Jurczakowski and G. Litwinienko, *J. Org. Chem.*, 2013, **78**, 9102-9112.
6. A. Staško, V. Brezová, S. Biskupič and V. Mišík, *Free Rad. Res.*, 2007, **41**, 379-390.
